# Structural insights into a citrate transporter that mediates aluminum tolerance in barley

**DOI:** 10.1073/pnas.2501933122

**Published:** 2025-08-05

**Authors:** Tran Nguyen Thao, Namiki Mitani-Ueno, Ryo Urano, Yasunori Saitoh, Peitong Wang, Naoki Yamaji, Jian-Ren Shen, Wataru Shinoda, Jian Feng Ma, Michihiro Suga

**Affiliations:** ^a^Degree Program in Interdisciplinary Sciences, Graduate School of Environmental, Life, Natural Science, and Technology, Okayama University, Okayama 700-8530, Japan; ^b^Research Core for Plant Stress Science, Institute of Plant Science and Resources, Okayama University, Kurashiki 710-0046, Japan; ^c^Division of Superconducting and Functional Materials, Research Institute for Interdisciplinary Science, Okayama University, Okayama 700-8530, Japan; ^d^Division of Photosynthesis and Structural Biology, Research Institute for Interdisciplinary Science, Okayama University, Okayama 700-8530, Japan; ^e^National Key Laboratory of Crop Genetics & Germplasm Enhancement and Utilization, College of Resources and Environmental Sciences, Nanjing Agricultural University, Nanjing 210095, China

**Keywords:** barley, aluminum resistance, membrane protein structure, citrate transporter, MATE transporter

## Abstract

*HvAACT1* is a major aluminum (Al)-tolerance gene in barley, encoding a citrate transporter that belongs to the multidrug and toxic compound extrusion (MATE) family. This transporter facilitates citrate secretion from the roots, thereby detoxifying external Al ions—a major constraint of crop production on acidic soils. In this study, we present the outward-facing crystal structure of HvAACT1, providing insights into a citrate transport mechanism. The putative citrate binding site consists of three basic residues—K126 in transmembrane helix 2 (TM2), R358 in TM7, and R535 in TM12—creating substantial positive charges in the C-lobe cavity. Proton coupling for substrate transport may involve two pairs of aspartate residues in the N-lobe cavity, one of which corresponds to the essential Asp pair found in prokaryotic H^+^-coupled MATE transporters belonging to the DinF subfamily. Structural coupling between proton uptake in the N-lobe and citrate extrusion in the C-lobe can be enabled by an extensive, unique hydrogen-bonding network at the extracellular half of the N-lobe. Mutation-based functional analysis, structural comparisons, molecular dynamics simulation, and phylogenic analysis suggest an evolutionary link between citrate MATE transporters and the DinF MATE subfamily. Our findings provide a solid structural basis for citrate transport by HvAACT1 in barley and contribute to a broader understanding of citrate transporter structures in other plant species.

## Significance

HvAACT1, belonging to the MATE family, transports citrate from the root cells to the rhizosphere, where it chelates toxic Al ions externally, thereby conferring plants’ Al tolerance. While most structurally characterized MATE proteins transport cationic or aromatic substrates, HvAACT1 is unique in transporting anionic substrates. It is crucial to elucidate the transport mechanism of anionic substrates to understand the functional versatility of MATE proteins. Our structure-based mutagenesis work identifies the citrate recognition and proton binding sites in HvAACT1, which are physically separated but functionally conjugated. These findings raise the possibility of a mechanism by which MATE proteins translocate negatively charged substrates, providing a solid structural basis for citrate efflux in plants and offering insights into Al tolerance mechanisms.

Soil acidification is a widespread issue that significantly impacts crop production worldwide (1–4). Approximately 30 to 40% of arable land is acidic, primarily due to soil leaching, organic matter decomposition, agricultural harvesting, and excessive nitrogen fertilizer use (1–4). When the soil pH drops below 5, aluminum (Al)—the most abundant metal in Earth’s crust—becomes soluble and highly toxic to plants. Al toxicity is considered as a major constraint of crop production on acidic soils. Highly reactive Al ions can bind to lipids, proteins, and nucleic acids, inhibiting root growth and, consequently, impairing overall plant development (5, 6). However, some plants have evolved mechanisms to tolerate Al toxicity. One such strategy involves secreting organic acids, such as citrate, malate, or oxalate, from root cells into the rhizosphere, where they chelate Al ions and mitigate their harmful effects (6–8).

Barley (*Hordeum vulgare*) is the fourth primary staple food cultivated worldwide but is relatively sensitive to Al toxicity compared with other cereal crops such as rice, rye, and wheat (9). Previous studies showed that there is a large natural variation in Al tolerance in barley, and the major gene controlling this variation is *HvAACT1* (Aluminum Activated Citrate Transporter), which encodes a citrate transporter belonging to the multidrug and toxic compound extrusion (MATE) protein family (10). *HvAACT1* is mainly and constitutively expressed in the roots (10), but upon Al exposure, HvAACT1 is activated to release citrate from the root tips for Al detoxification. Furthermore, the natural variation in Al tolerance was associated with the expression level of *HvAACT1* due to a 1-kb insertion of the CACTA-like transposon upstream of the *HvAACT1* coding region (11). Evolutionary analysis showed that the insertion is acquired during the expansion of barley cultivation onto the acidic soils in the Far-Eastern region. Recently, it was reported that introgression of this 1-kb transposon into an elite malting cultivar resulted in two to three times more grain yield compared with the original cultivar when grown on acidic soil (12), indicating the importance of this transporter gene in barley production on acidic soil. However, the structural information of HvAACT1 is unknown.

The MATE family, found across all domains of life, has been categorized into three subfamilies based on sequence similarity: two prokaryotic subfamilies called NorM and DinF, and an eukaryotic subfamily eMATE (13). Most MATE proteins are expressed in the plasma membrane, working to pump substrates out of the cell by coupling with a proton (H^+^) or cation (Na^+^) electrochemical gradient, but show diversity in transport substrates. Prokaryotic MATE transporters exhibit substrate promiscuity, allowing them to extrude multiple antimicrobial agents from the cell. This property confers multidrug resistance to pathogens, making MATE transporters critical pharmacological targets. Unlike prokaryotic MATE transporters, most plant eMATE transporters have higher substrate specificity (14) and participate in various biological processes, including xenobiotic efflux (15), accumulation of flavonoids and alkaloids (15–18), translocation of ions (15), hormone signaling (19–21), iron homeostasis, and aluminum detoxification (11, 22, 23). Thus, individual plant genomes often carry a substantially more significant number of MATE genes than those in bacterial genomes. For instance, *Oryza sativa*, *Arabidopsis thaliana*, *Solanum tuberosum*, and *Glycine max* have 45, 56, 64, and 117 MATE genes, respectively (24–26), highlighting the significance of the MATE family in the plant kingdom.

The structures of MATE transporters from all three subfamilies have been determined (27–34). In general, MATE transporters share a common topology consisting of 12 transmembrane helices (TM), which form quasisymmetric lobes: the N-lobe (TM1-TM6) and the C-lobe (TM7-TM12). These lobes create a central cavity that can be accessed from either the intracellular side (inward-facing conformation) or the extracellular side (outward-facing conformation). This process follows the alternating access mechanism, where transporters switch between conformations to translocate substrates across the membrane (34). Most reported MATE structures adopt an outward-facing conformation, with the inward-facing structure of PfMATE derived from *Pyrococcus furiosus* being a rare exception (34). Despite their shared structural topology, MATE transporters from different subfamilies exhibit divergent transport properties. NorM MATEs transport substrates by interacting with cations via an acidic residue in the C-lobe cavity (28, 31), whereas Dinf MATEs rely on conserved acidic residues in the N-lobe cavity for proton-coupled substrate transport (29, 30, 35). Structural studies of several plant MATE transporters, including AtDTX14 from *Arabidopsis thaliana*, CasMATE from *Camelina sativa*, and NtMATE2 from *Nicotiana tabacum*, suggest that eMATE transporters function similarly to NorM MATEs, utilizing conserved acidic residues in the C-lobe cavity for proton and substrate transport (32, 33, 36). However, these acidic residues are absent in citrate-transporting MATE transporters such as HvAACT1, indicating a different substrate binding site and energy coupling mechamism.

A common feature of substrate-binding sites in structurally characterized MATE transporters is the presence of acidic residues and surrounding hydrophobic side chains, which interact with cationic or uncharged aromatic substrates. The binding sites of protons or Na^+^ ions, serving as secondary substrates, are typically shared with or located near the primary substrate-binding site. The antiport mechanism involves direct competition for shared sites or local structural changes induced by secondary substrate binding, which influence the primary site and alter substrate affinity through allosteric effects. However, the transport mechanism for anionic substrates like citrate cannot be inferred from existing MATE structures due to the opposing charges between the primary and secondary substrates.

Here, we present the crystal structure of HvAACT1, a citrate MATE transporter from barley (*Hordeum vulgare*), in its outward-facing conformation at 3.2 Å resolution. For comparison, we predicted the inward-facing HvAACT1 structure by AlphaFold2 (37). Our structural, functional, and computational analyses provide insights into the citrate binding site and its transport mechanism of this unique plant MATE group, which differs from NorM-like eMATEs. Furthermore, we identified potential proton-coupling sites in HvAACT1, similar to those in PfMATE transporters of the DinF subfamily. These findings strongly support an evolutionary link between the plant eMATE3 group, including HvAACT1, and the prokaryotic DinF MATE subfamily.

## Results

### Phylogenetic Characterization and Structural Determination of HvAACT1.

Phylogenetic analysis of MATE transporters indicates that citrate-transporting MATEs, such as HvAACT1 and OsFRDL1 from rice, belong to a distinct eMATE subfamily (eMATE3). This finding aligns with previous observations regarding their unique gene structures and protein motifs (24, 38). We refer to the eMATE3 group as DinF-like Carboxylic Acid-Translocating eMATEs (CAT-eMATEs) due to their close evolutionary relationship with the DinF subfamily, while other eMATE groups (eMATE1, eMATE2, and eMATE4) are more closely related to the NorM subfamily (*SI Appendix*, Fig. S1).

Attempts to crystallize full-length HvAACT1 were unsuccessful, likely due to disordered regions. Secondary structure prediction revealed a long N-terminus and an extended intracellular loop between TM2 and TM3 (L2-3). Removing these regions (Δ1-56, Δ153-199) and introducing five-point mutations—C351A, C388L, T495V to enhance thermostability, along with R105H, R456S to reduce surface entropy—successfully enabled crystallization of a modified construct, hereinafter referred to as HvAACT1_cryst_. None of these mutations exists in conserved regions among citrate MATEs. Functional assays in *Xenopus* oocytes confirmed that HvAACT1_cryst_ retains citrate efflux activity comparable to the wild type (*SI Appendix*, Fig. S2), suggesting that the truncated N-terminal and L2-3 loop regions are not essential for citrate transport. The HvAACT1_cryst_ crystals diffracted to 3.2 Å resolution in the best direction, though the diffraction patterns exhibited severe anisotropy. The diffraction data were processed using the Diffraction Anisotropy Server (39), allowing us to solve the crystal structure of HvAACT1_cryst_ at 3.2 Å resolution (*SI Appendix*, Table S1). For comparison, we predicted an inward-facing HvAACT1_cryst_ structure with the truncated sequence using AlphaFold2 (*Materials and Methods*) (37).

### The Overall Structure of HvAACT1_cryst_.

**Fig. 1.**
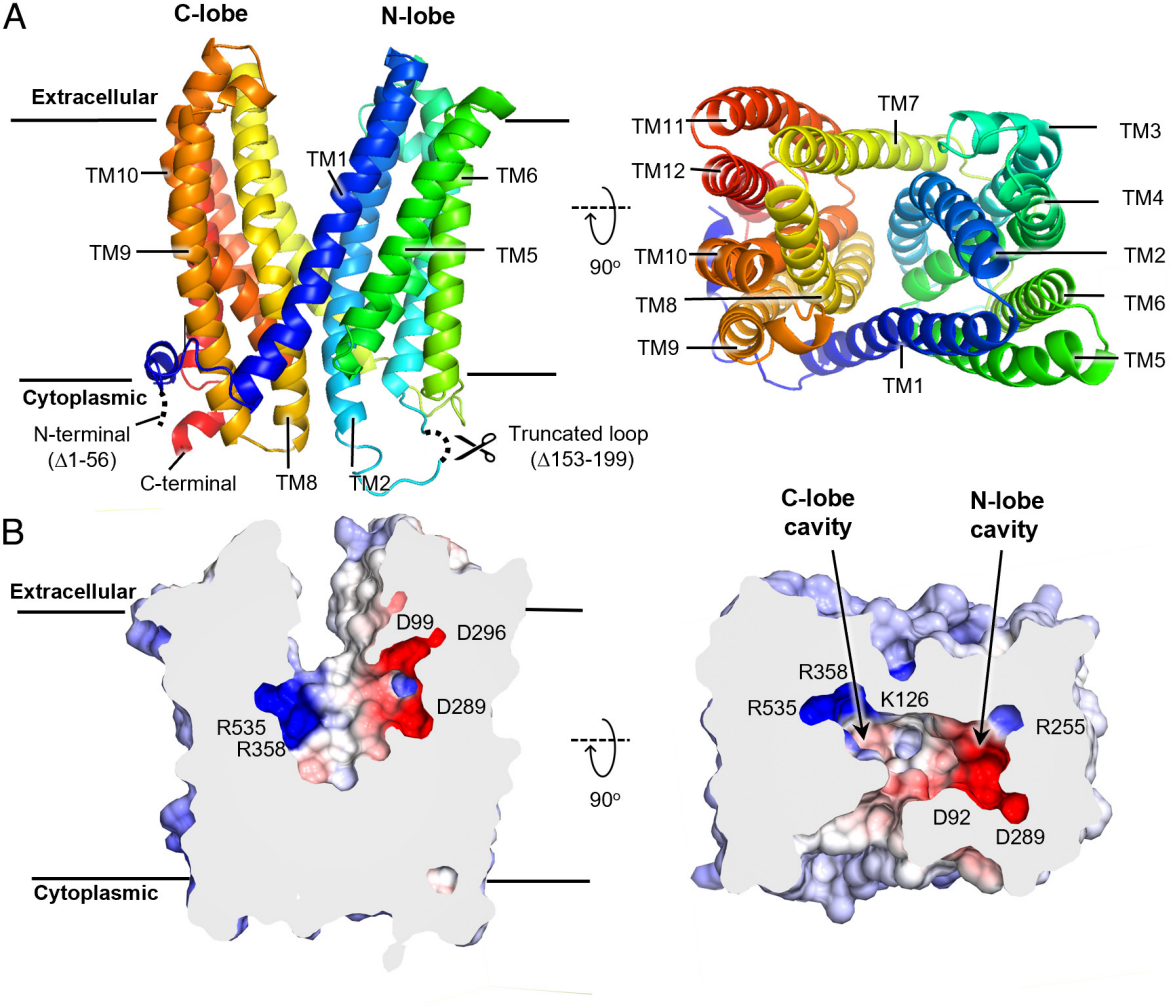
The overall crystal structure of HvAACT1_cryst_. (*A*) Cartoon representations of the crystal structure of HvAACT1^_cryst_^ in its outward-facing conformation, viewed from the membrane plane (*Left*) and the apoplast (*Right)*. (*B*) Surface representations of the transporter are sliced through the center of the inner cavity in the side (*Left*) and *Top* (*Right*) orientations. Acidic and basic residues contributing to the surface charge are labeled. The transporter surface is colored according to the electrostatic surface potential, calculated using the APBS software package in the CueMol2. The electrostatic potential is displayed in SA mode, ranging from −12 kT/e (red, most anionic) to +12 kT/e (blue, most cationic), with neutral regions in white.

**Fig. 2.**
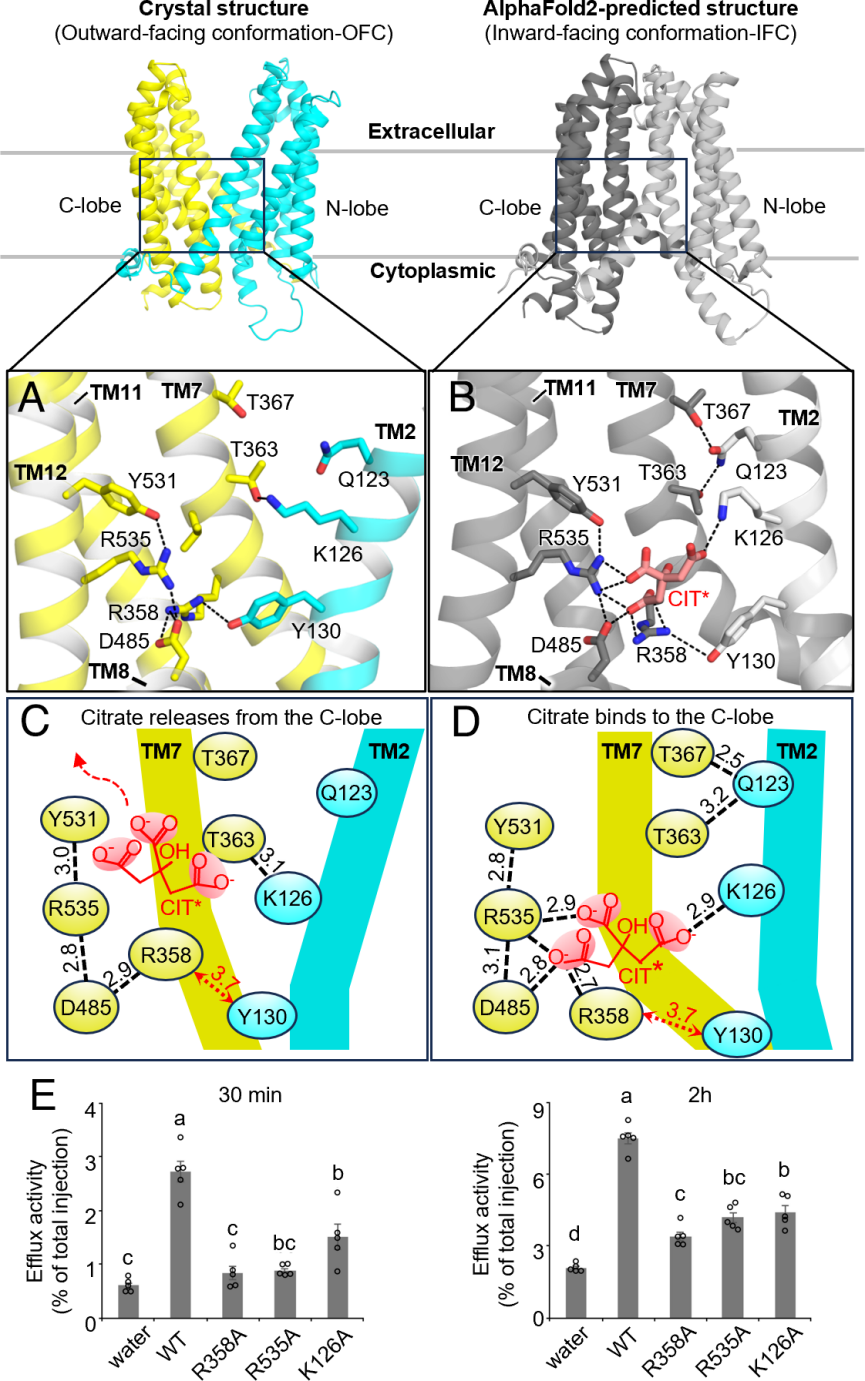
Putative citrate binding site in the C-lobe cavity. (*A* and *B*) Close-up views of the putative citrate binding site in (*A*) the crystal structure of HvAACT1_cryst_ and (*B*) the AlphaFold2-predicted inward-facing structure of HvAACT1_cryst_ with modeled citrate (CIT*). (*C* and *D*) Two-dimensional diagrams illustrating the interaction between three basic residues in the C-lobe cavity and citrate in (*C*) the outward-facing conformation and (*D*) the predicted inward-facing conformation. Hydrogen bonds and salt bridge interactions are represented by dashed black lines, with the distance between residues and citrate shown in angstroms. (*E*) Citrate efflux activity of HvAACT1 and its mutants expressed in *Xenopus* oocytes. The release of ^14^C-labeled citrate was measured after 30 min (*Left*) and 2 h later (*Right*). Data are presented as means ± SEM (*n* = 5, each replicate containing 4 to 6 oocytes). According to Tukey–Kramer’s test, different letters indicate significant differences (*P* < 0.05).

The crystal structure of HvAACT1_cryst_ adopts a typical V-shaped outward-facing conformation, similar to the previously reported MATE transporters (Fig. 1*A*). Structural comparisons between HvAACT1_cryst_ and other outward-facing structures revealed minor carbon-alpha RMS deviations ranging from 1.90 to 4.08 Å, whereas the inward-facing structure exhibited a larger deviation of 6.54 Å (*SI Appendix*, Fig. S3 and Table S2). The twelve transmembrane helices of HvAACT1_cryst_ form two distinct lobes: N-lobe (TM1-TM6) and C-lobe (TM7-TM12). These lobes make tight contacts on the intracellular side, sealing the transporter’s internal cavity from the cytoplasm for approximately 15 Å (Fig. 1*B*). These contacts include two salt bridge interactions: one between E77^TM1^ and K276^L4-5^/K420^TM9^ and another between E142^TM2^ and R347^TM7^, reinforced by Q492^L10-11^ (*SI Appendix*, Fig. S4). Additionally, extensive hydrogen bond clusters stabilize the structure, involving S138^TM2^ and the carbonyl of S134^TM2^ from the N-lobe, interacting with Q406^TM8^, Q492^L10-11^, and the carbonyl of F489^TM10^ from the C-lobe (*SI Appendix*, Fig. S4). The N-lobe cavity is concave primarily in the middle region of the membrane, and its apex extends laterally (Fig. 1*B*). The electrostatic surface potential of the inner cavity in HvAACT1_cryst_ reveals a positively charged C-lobe, attributed to three basic residues: K126^TM2^, R358^TM7^, and R535^TM12^. Notably, K126^TM2^ belongs to TM2 but extends into the C-lobe, where it forms a hydrogen bond with T363^TM7^, contributing to the positive charge of the C-lobe (Fig. 2*A*). Conversely, the N-lobe cavity is primarily negatively charged, owning to a cluster of four Asp residues (D92^TM1^, D99^TM1^, D289^TM5^, and D296^TM5^) (Fig. 1*B*). This charge distribution differs from NorM-like eMATE transporters, such as CasMATE and AtDTX14, which exhibit negatively charged cavities in both lobes (*SI Appendix*, Fig. S5). This distinct charge pattern highlights a unique feature of citrate transporters among plant MATE transporters.

### Putative Citrate Binding Site.

Despite 0.1 M citrate in the crystallization buffer, no electron density corresponding to citrate was detected. Given that the HvAACT1_cryst_ structure adopts an outward-facing conformation, citrate release may be facilitated, likely explaining the absence of a bound substrate in the crystal structure. Based on the electrostatic surface potential of the cavity, we hypothesized that citrate binds to the positively charged cavity in the C-lobe. Among the three basic residues of this cavity—K126^TM2^, R358^TM7^, and R535^TM12^—both Arg residues are fully conserved, while K126^TM2^ is conserved as either Lys or Arg in citrate MATEs (*SI Appendix*, Figs. S6 and S7). The orientation of K126^TM2^ is stabilized by a hydrogen bond with T363^TM7^ (Fig. 2*A*), while R535^TM12^ is fixed by tight interactions with D485^TM8^ and Y531^TM12^ (Fig. 2*A*). Additionally, R358^TM7^, located deep in the C-lobe, is buried by Y130^TM2^ protruding from TM2 and forms a salt bridge with D485^TM10^ (Fig. 2*A*). These three basic residues may form a recognition pocket for citrate, which exists predominantly in its trivalent form (citrate^3-^) in the cytoplasm due to its p*K*a values of 3.1, 4.8, and 6.4 (40). When citrate is placed within this positively charged pocket, it experiences only minor steric hindrance and can be accommodated with slight side-chain adjustments. For example, flipping the K126^TM2^ side-chain away would eliminate its hydrogen bond with T363^TM7^, creating additional space for citrate binding. Interestingly, HvAACT1 does not transport malate, which lacks one carboxymethyl group compared to citrate (10). When manually placed in the binding site, malate interacts only with two basic residues, suggesting that all three are required for citrate specificity. Ala-substituted mutants (K126A, R358A, and R535A) were functionally analyzed in *Xenopus* oocytes to confirm the importance of these basic residues. Although all mutants exhibited reduced citrate efflux without a complete loss of activity, we repeated the transport assay to confirm this and obtained similar results (Fig. 2*E* and *SI Appendix*, Fig. S19). These results indicate that K126^TM2^, R358^TM7^, and R535^TM12^ are critical for HvAACT1 function, suggesting their role in citrate recognition and transport.

### Putative Proton-Binding Sites and Hydrogen Bond Network in the N-Lobe Cavity.

**Fig. 3.**
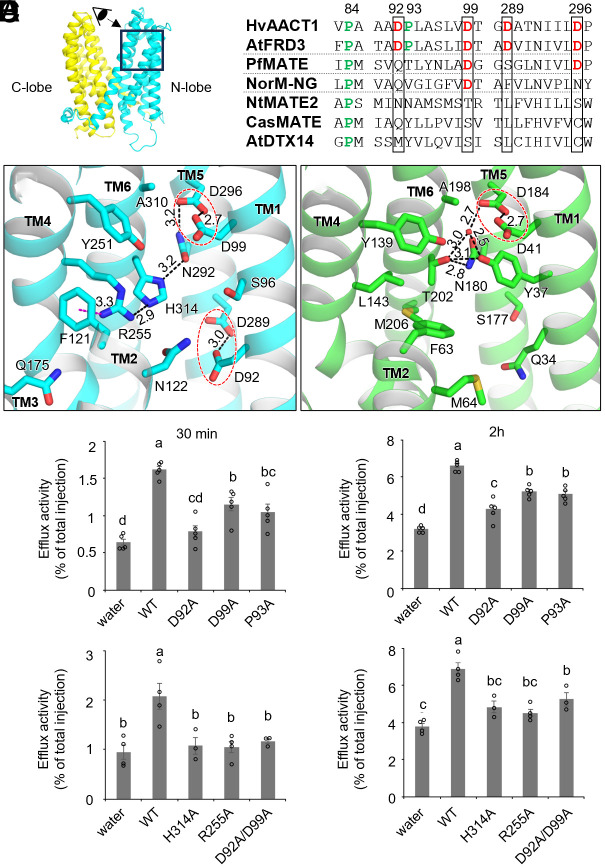
Two possible protonation sites in the N-lobe cavity. (*A*) Partial sequence alignment highlighting the two Asp pairs (D92, D99, D289, and D296) and two Pro residues (P84 and P93) found in the HvAACT1_cryst_ structure. (*B* and *C*) Close-up views of possible protonation sites: (*B*) two protonation sites in the N-lobe cavity of HvAACT1_cryst_ (cyan) and (*C*) a single protonation site in the N-lobe cavity of PfMATE (PDB ID: 3VVN, green). Hydrogen bond and cation–π interactions are represented by dashed black and magenta lines, respectively. Distances between two Asp residues and the hydrogen bonding network are shown in angstrom. A water molecule is depicted as a red sphere. The cartoon’s inset indicates the view directions for (*B*) and (*C*). (*D* and *E*) Citrate efflux activity of HvAACT1 and its mutants expressed in *Xenopus* oocytes assessing (*D*) potential protonation sites, including a newly identified Pro at TM1, and (*E*) the extensive hydrogen bond network in the N-lobe cavity. The release of ^14^C-labeled citrate was measured after 30 min (*Left*) and 2 h (*Right*). Data are presented as means ± SEM (*n*=3 to 5, with each replicate containing 4 to 7 oocytes). According to Tukey–Kramer’s test, different letters indicate significant differences (*P* < 0.05).

MATE transporters from the eMATE and DinF subfamilies utilize a proton gradient to drive substrate export. However, their protonation sites differ: NorM-like eMATEs utilize acidic residues in the C-lobe cavity, and DinF MATEs rely on acidic residues in the N-lobe cavity. Unlike NorM-like eMATEs, the C-lobe cavity of HvAACT1_cryst_ is positively charged (Fig. 1*B* and *SI Appendix*, Fig. S5) and lacks acidic residues for proton binding (*SI Appendix*, Figs. S6 and S8). In contrast, the N-lobe of HvAACT1_cryst_ contains a cluster of acidic residues, two of which—D99^TM1^ and D296^TM5^—are identical to the essential proton binding Asp residues in DinF MATEs (Fig. 3 *A* and *B* and *SI Appendix*, Figs. S6 and S8). Specifically, D99^TM1^ and D296^TM5^ of HvAACT1_cryst_ correspond to D41^TM1^ and D184^TM5^ in PfMATE from the DinF subfamily. In the HvAACT1_cryst_ structure, D99^TM1^ forms a carboxyl–carboxylate interaction with D296^TM5^ at a distance of 2.7 Å, similar to the D41^TM1^/D184^TM5^ pair in PfMATE (29). This interaction suggests that at least one Asp residue is protonated, allowing it to share a hydrogen bond with the other. Uniquely, HvAACT1 contains an additional Asp pair, D92^TM1^/D289^TM5^, positioned closer to the cytoplasmic side from the D99^TM1^/D296^TM5^ pair, with a 3.0 Å interaction distance (Fig. 3*B*). The two Asp pairs are highly conserved among citrate MATE transporters: D92, D99, and D296 are fully conserved, while Tyr replaces D289 in *Vigna umbellata* MATE1 and *Medicago truncatula* MATE69. Functional analysis showed that both D92A and D99A mutations reduced citrate efflux in the oocytes (Fig. 3*D*), suggesting that either one or both Asp pairs act as protonation sites in HvAACT1, even though the D92A/D99A double mutant retained residual transport activity (Fig. 3*E*).

Structural studies on DinF MATEs indicate that deprotonation/protonation of the Asp at TM1 (D41 in PfMATE) triggers rearrangements in a water-mediated hydrogen bond network. This network, involving D41^TM1^, Y37^TM1^, Y139^TM4^, N180^TM5^, D184^TM5^, and T202^TM6^ within the N-lobe of PfMATE (Fig. 3*C*), facilitates TM1 to shift between “straight” and “bend” forms, a key step in switching between outward- and inward-facing conformations (29, 34). Among these six key residues in PfMATE, four residues are also conserved in citrate MATE transporters, equivalent to D99^TM1^, Y251^TM4^, N292^TM5^, and D296^TM5^ in HvAACT1 (Fig. 3*B*). Notably, T202^TM6^ in PfMATE is absent in HvAACT1 but is replaced by H314^TM6^, which contributes to a similar bonding network along with an extended hydrogen bond network unique to HvAACT1 (Fig. 3*B*). A distinctive feature of HvAACT1_cryst_ is R255^TM4^, which is sandwiched by F121^TM2^ and H314^TM6^, forming a cation–π interaction with F121^TM2^, and hydrogen bond with H314^TM6^, and likely a water-mediated hydrogen bond with N122^TM2^ and Q175^TM3^. This network extends to K126^TM2^, a residue implicated in citrate binding (*SI Appendix*, Fig. S9). Interestingly, PfMATE lacks equivalents to R255^TM4^ and H314^TM6^, where hydrophobic residues fill the space instead. Mutants R255A and H314A were examined in *Xenopus* oocytes to test their functional significance. Both mutations significantly reduced citrate efflux (Fig. 3*E*), highlighting the involvement of the hydrogen bond network in HvAACT1 function. These findings suggest that the protonation site in the N-lobe is structurally linked to the citrate-binding site in the C-lobe, enabling efficient transport.

### Structural Elements Governing Helix Flexibility and the Inward-Facing Conformation.

Structural analysis of PfMATE has shown that the transition between outward-facing and inward-facing conformations involves the rigid body movements of TM bundles—TM2-6 in the N-lobe and TM8-12 in the C-lobe—as well as a shift in TM1 and TM7 from a “straight” to a “bent” conformation (34). This flexibility in TM1 and TM7 is crucial for the alternating access mechanism in many MATE transporters (28–30, 32). In the HvAACT1_cryst_ structure, TM1 and TM7 appear straight, unlike the “bent” TM1 in PfMATE (29) and TM7 in the NorM-like eMATEs AtDTX14 (32) (*SI Appendix*, Fig. S10). To investigate what allows these helices to bend, we used AlphaFold2 to predict the inward-facing conformation and compared it to the outward-facing structure (*Materials and Methods*) (37). The RMSD between these conformations was 5.1 Å, but when comparing the N-lobe and C-lobe separately, the RMSD values dropped to 0.6 Å and 0.5 Å, respectively. Areas with large RMSD values located at the N-terminal halves of TM1 and TM7 when the N- or C-lobes of the outward-facing crystal structure and the inward-facing predicted structure of HvAACT1_cryst_ are superimposed, with the N-terminal halves of TM1 and TM7 predicted to bend 30 and 35 degrees, respectively, in the inward-facing structure (*SI Appendix*, Fig. S11). These findings suggest that HvAACT1 uses a similar alternating access mechanism as PfMATE.

Intriguingly, TM1 and TM7 bend at D92^TM1^ and R358^TM7^ in the inward-facing conformation (*SI Appendix*, Fig. S11). Since D92^TM1^ in TM1 is the candidate protonation site, and R358^TM7^ is part of the substrate-binding site, proton or citrate binding may locally induce the bending of TM1 and TM7, triggering the transition between the outward- and inward-facing conformations. To explore additional structural elements enabling this flexibility, we examined proline residues, which are known to induce bends in helices (41). We identified two proline residues in TM1, P84^TM1^ and P93^TM1^, that might facilitate structural changes. P84^TM1^ is equivalent to P29^TM1^ in PfMATE, a residue responsible for bending TM1 in PfMATE. In contrast, P93^TM1^ is unique to citrate MATE transporters and located next to D92^TM1^, the kinking site in the predicted inward-facing structure (*SI Appendix*, Fig. S11), and the potential protonation site. These prolines lack main chain hydrogen bond interactions and disrupt the secondary structure of TM1 in HvAACT1_cryst_ (*SI Appendix*, Fig. S12). The P93A mutation reduced the citrate transport activities in oocytes (Fig. 3*D*), suggesting that P93^TM1^ likely contributes to the bending of TM1. Unlike TM1, TM7 does not contain proline residues, but R347^TM7^ constitutes the intracellular contacts with E142^TM2^ and Q492^L10-11^ in the HvAACT1_cryst_ structure (*SI Appendix*, Fig. S4). Breaking these interactions during the transition may cause TM7 to bend, facilitating the switch between conformations.

Based on the predicted inward-facing structure, we examined the putative substrate-binding site. In the predicted inward-facing structure, two pairs of helices at the lobe interface, TM1–TM2 and TM7–TM8, move closer together at the extracellular side to seal the gate, while they separate to create a wider opening at the intracellular side (*SI Appendix*, Fig. S11). This transition expands the substrate-binding site, which is supposed to allow citrate to bind in the inward-facing conformation. Due to the shift of these helices, the binding site, composed of K126^TM2^, R358^TM7^, and R535^TM12^, slightly expands. This expansion removes steric hindrance with citrate, which is present in the outward-facing state, explaining why the inward-facing state has a higher substrate affinity (Fig. 2*B*). Notably, in the outward-facing conformation, K126^TM2^ is stabilized by a hydrogen bond with T363^TM7^. However, in the predicted inward-facing conformation, K126^TM2^ loses this interaction, and T363^TM7^ instead forms a hydrogen bond with Q123^TM2^ (Fig. 2*B*). It should be noted that the AlphaFold2-predicted structure is inherently speculative, and substrate-bound and occluded forms are required to decipher the transport mechanism. Nevertheless, we performed molecular dynamics simulations (MD) to examine the substrate binding in the inward-facing state, in which K126^TM2^ was identified as a key residue that consistently interacts with citrate across three binding sites. One site involves R255^TM4^, another involves R273^TM4^, and the third involves R358^TM7^ and R535^TM12^, corresponding to the putative binding site (*SI Appendix*, Figs. S14–S18). These results support our proposal but also raise the possibility that K126^TM2^ may form alternative binding sites with R255^TM4^ or R273^TM4^. This possibility may be plausible, as R255^TM4^ is located near the secondary substrate-binding sites, whereas R273^TM4^ may function as a transient uptake site due to its position at the intracellular vestibule. Furthermore, the R255^TM4^ mutation led to decreased citrate transport activity (Fig. 3*E*).

## Discussion

**Fig. 4.**
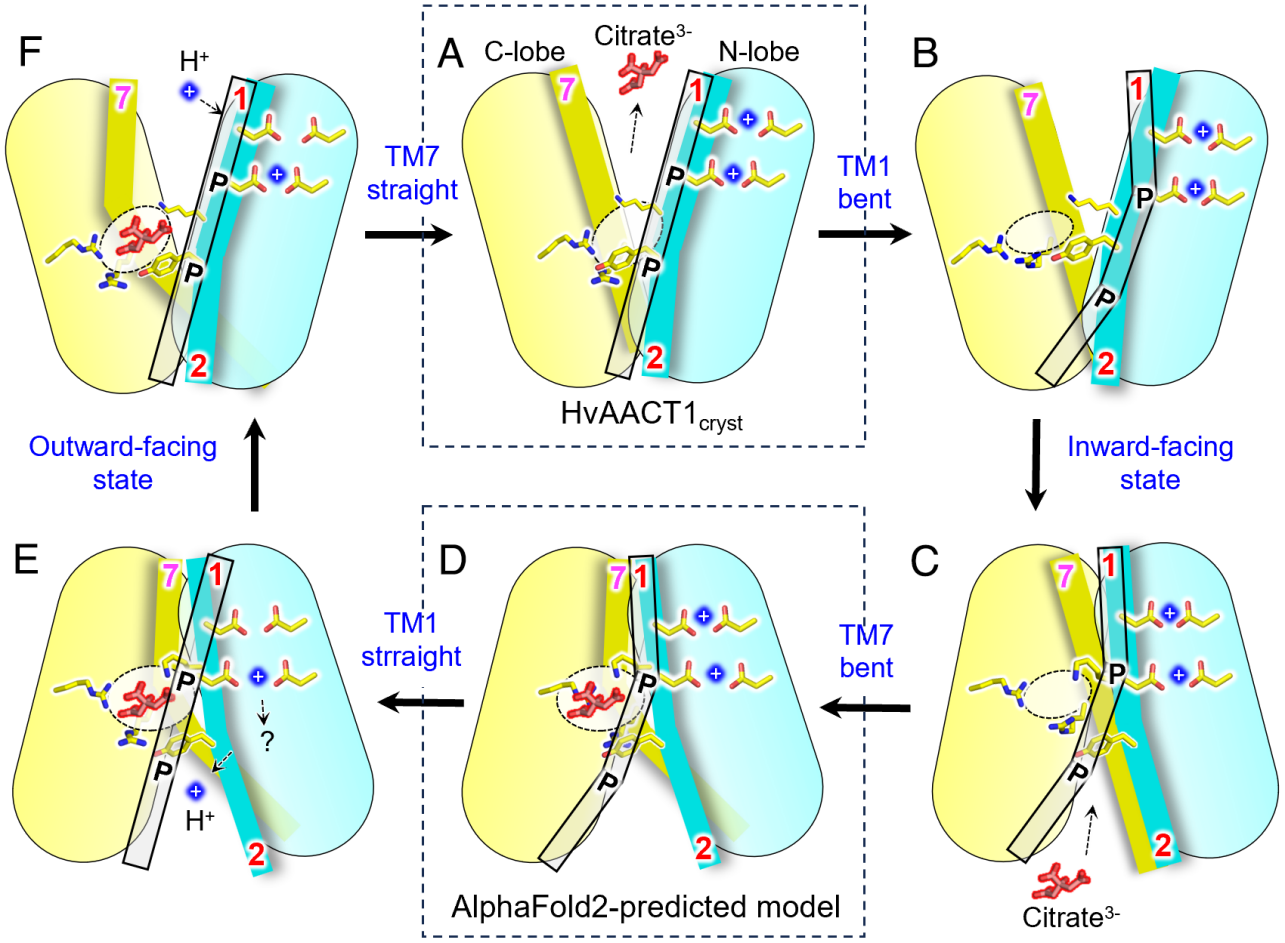
Proposed transport mechanism of HvAACT1. (*A*) In the outward-facing state (this study), substrate release is facilitated by the acidic pH on the extracellular side, which protonates Asp residues in the N-lobe cavity. This protonation triggers changes in the hydrogen-bonding network within the N-lobe cavity, involving one or both Asp pairs. (*B*) These changes lead to the bending of TM1 at P84 and P93, initiating structural transition. (*C*) TM1 bending induces a switch from the outward-facing to the inward-facing state. In this transition, the position of TM2 relative to the C-lobe shifts, and Y130 from TM2 moves away from the substrate-binding site, allowing TM7 to bend. (*D*) This movement shifts R358 and expands the substrate-binding site, enabling citrate to bind. (*E*) In the inward-facing conformation, at least one of the Asp pairs becomes deprotonated, releasing protons into the cytoplasm and reversing the bending of TM1. One Asp residue may remain protonated. (*F*) The transporter returns to equilibrium, switching from the inward-facing to the outward-facing state. Once citrate is released to the extracellular side, and protons bind to the N-lobe cavity, Y130 reinserts into the C-lobe cavity, forcing TM7 to straighten, restoring the outward-facing state. Key TM helices—TM1 (transparent), TM2 (cyan), and TM7 (yellow)—are displayed as sticks with their numeric labels.

In this study, we determined the structure of the citrate efflux transporter HvAACT1 in its outward-facing conformation, which adopts a typical V-shaped MATE structure. This transporter contains two Asp pairs (D92^TM1^/D289^TM5^ and D99^TM1^/D296^TM5^) in the N-lobe cavity and three basic residues (K126^TM2^, R358^TM7^, and R535^TM12^) in the C-lobe. All these residues are critical for citrate efflux, as mutations in any of them significantly reduce transport activity (Figs. 2 and 3 and *SI Appendix*, Fig. S19). Nevertheless, transport activity was still retained in these mutants, which may result from the alternative use of citrate in its divalent form. To explain the possible coupling mechanism between the putative proton binding site in the N-lobe and substrate binding site in the C-lobe, we propose a model for citrate efflux (Fig. 4). Based on the sequence and structural similarity between HvAACT1 and PfMATE, we speculate that the two Asp pairs serve as potential protonation sites and that the protonation-induced structural changes in HvAACT1 resemble those in PfMATE. In the outward-facing conformation, protonation of either or both Asp pairs likely reorganizes the tight hydrogen bonding network in the N-lobe cavity, leading to the bending of TM1, possibly near P84^TM1^ and P93^TM1^ (Fig. 4*B*). Notably, the bonding network in the N-lobe cavity of HvAACT1 is more extended than that in PfMATE, spanning from the protonation sites in TM1 across multiple TMs in the N-lobe cavity to TM2, which contributes to the substrate binding site via K126^TM2^ and Y130^TM2^. This extended connection may couple protonation in the N-lobe with regulation of substrate binding affinity in the C-lobe. The bending of TM1 is likely a key event that drives the conformational switch of HvAACT1 from an outward-facing to an inward-facing state. Upon adopting the inward-facing conformation, Y130 ^TM2^ likely retracts from the C-lobe, expanding the binding pocket (Fig. 4*C*). Once expanded, this pocket may accommodate citrate^3-^ at the center of a positively charged triad formed by K126^TM2^, R358^TM7^, and R535^TM12^ in the C-lobe cavity (Fig. 4*D*). The protonated Asp residues may then release protons (H^+^) into the cytoplasm, where the pH is higher. This deprotonation in the N-lobe, together with the substrate binding in the C-lobe, could trigger a structural transition back to the outward-facing conformation (Fig. 4*E*). During this transition, Y130^TM2^ may be reinserted into the C-lobe cavity, reducing the size of the citrate binding site. The insertion of Y130^TM2^ may also partially shield R358^TM7^, lowering the electrostatic potential at the substrate binding site and facilitating citrate release in the outward-facing state. Additionally, citrate^3-^ is likely protonated to become Hcitrate^2-^ by the acidic pH of the extracellular apoplastic space or chelated with Al to form a neutral complex. As a result, citrate is released from the binding site, and HvAACT1 returns to its substrate-free state, ready for a new transport cycle (Fig. 4*A*).

Members of the DinF subfamily, such as PfMATE, typically contain a single Asp pair in the N-lobe cavity for proton coupling, whereas HvAACT1 requires two Asp pairs for functionality. One notable feature is that the predicted p*K*_a_ of D92^TM1^ (p*K*_a_ = 2.5) is significantly lower than that of D99^TM1^ (p*K*_a_ = 5.7) (*SI Appendix*, Table S3). This suggests that the D99/D296 pair is the primary protonation site at neutral pH in the outward-facing state, while the D92/D289 pair may play a structural role rather than acting as a protonation site. However, we cannot rule out the possibility that D92/D289 also participates in proton coupling or relay during transport. Moreover, both Asp pairs (D99/D296 and D92/D289) may be protonated simultaneously, depending on the environmental pH or transport cycle stage. Such dual protonation events could trigger more robust conformational changes required for citrate transport. Surprisingly, single mutations in either Asp pair significantly reduced transport activity, but double mutations did not wholly abolish citrate extrusion (Fig. 3 *B* and *C*). This raises the possibility that the proton motive force is not the sole energy source for citrate efflux. Instead, HvAACT1 may utilize an alternative alkaline ion, similar to ClbM, which couples transport to Na^+^ or K^+^ (42, 43). Indeed, a citrate MATE homolog, SbMATE from *Sorghum bicolor*, can use Na^+^ and K^+^, in addition to H^+^, for citrate extrusion (44). Further investigation is needed to determine whether other citrate MATE transporters exhibit similar promiscuity in ion coupling.

The cryoelectron microscopy structure of AtALMT1, an Al-activated malate transporter involved in Al tolerance, was recently analyzed (45, 46). This transporter has a distinct anion channel structure, where TM1-3 and TM4-6 form a malate-permeation pathway with pseudo-two-fold symmetry. Two Arg pairs at the center of the pore mediate malate recognition. The pore diameter in the open state is approximately 5 Å, allowing malate to pass but not citrate. An anion channel-type mechanism is better suited for transporting malate than a transporter-type one, as malate is smaller, carries fewer total charges, and exhibits a two-fold symmetry, enabling recognition by Arg pairs. In contrast, citrate lacks three-fold symmetry; indeed, HvAACT1 has an asymmetric citrate binding site. This suggests that a transporter mechanism, rather than a channel mechanism, was evolutionarily selected for citrate transport. ALMTs are known to be activated by Al. AtALMT1 binds Al to a site formed by three acidic residues and two acetates, including a conformational change that opens extracellular pores in TM5 and TM6. This structural change is thought to be the mechanism underlying Al activation. However, HvAACT1 likely follows a different Al activation mechanism, lacking a similar acidic core. Based on the outward-facing structure of HvAACT1_cryst_, one possibility is that Al^3+^ binding near the substrate site facilitates substrate release from the transporter.

Phylogenetic analysis of MATE transporters shows that the CAT-eMATE group, which includes citrate MATE transporters, is closely related to the DinF subfamily but distinct from other eMATEs (*SI Appendix*, Fig. S1). Structural comparison reveals that the cavity of HvAACT1_cryst_ differs entirely from that of the NorM-like eMATEs, suggesting that citrate transporters employ a different substrate binding site and energy coupling mechanism. However, the presence of a critical Asp pair for proton coupling in the N-lobe cavity of HvAACT1_cryst_ links citrate transporters evolutionarily to the DinF subfamily. While DinF MATEs primarily transport cationic and aromatic compounds, their positively charged cavities may also interact with negatively charged lipids, as suggested by the MD simulations of PfMATE (34). Thus, plant CAT-eMATE transporters may have evolved from the DinF-like ancestors under selective pressure to transport negatively charged substrates. Both DinF MATEs and CAT-eMATEs belong to the same superfamily as MurJ and Wzx flippases, members of the multidrug/oligosaccharidyl-lipid/polysaccharide (MOP) superfamily. Studies on these flippases have highlighted the role of conserved basic residues and cationic pockets in recognizing negatively charged lipids (47, 48). This raises the possibility that the CAT-eMATE transporters and DinF subfamily share a common prokaryotic ancestor specialized in lipid and anionic metabolite transport. Indeed, past research on MATE transporters has focused mainly on their role in multidrug resistance, which may have overshadowed their actual substrate specificities. As a result, the diversity of transport mechanisms used by MATE transporters for differentially charged substrates has likely been overlooked.

In conclusion, this study provides a structural framework for understanding substrate recognition and transport in citrate MATE transporters. It also offers insights into their evolutionary origins, shedding light on the diverse functional roles of plant MATE transporters. These findings serve as a valuable reference for studying citrate transporters in other plant species.

## Materials and Methods

### Sequence Alignment and Phylogenetic Tree Generation.

Protein sequences of 94 MATE transporters were aligned by ClustalW with the gap opening and gap extension penalties of 10 and 0.2, respectively. A phylogenetic tree was then constructed using the Maximum Likelihood method based on the best-fit substitution model (LG+F) with 1,000 bootstrap replicates. Positions with less than 50% site coverage, where more than 50% of sequences contained gaps or missing data, were excluded from the analysis. Sequence alignment and evolutionary analyses were conducted in MEGA 12 (49), and the phylogenetic tree was visualized using iTOL (https://itol.embl.de/).

### Protein Expression and Membrane Extraction.

The cDNA of *HvAACT1* was fused with EGFP and a double hexa-histidine tag on its C-terminal, then cloned into the pFastBac1 vector for baculovirus expression in Sf9 cells. A sequence of a TEV protease cleavage site was added right after *HvAACT1* for subsequent removal of the EGFP tag during purification. Mutant constructs were created using the PrimeSTAR Mutagenesis Basal Kit (Takara). All constructs were transfected into insect Sf9 cells by the Baculovirus expression system using CellFectin^®^ II reagent (Thermo Fisher Scientific). Insect Sf9 cells were transfected with P3 viruses for large-scale protein expression. After 72 h of incubation at 27 °C, infected cells were harvested by centrifugation at 10,000×*g* for 15 min, then resuspended and disrupted by an ultrasonic disruptor UD-211 (TOMY) for 10 min in the presence of 5 µM protease inhibitor E-64. All following experiments were performed on ice or in a cold room (~7 °C) to ensure protein stability unless otherwise noted. The crude membrane was collected by ultracentrifugation at 150,000×*g* for 2 h. The pellet was homogenized in a buffer containing 20 mM Tris-HCl pH 7.5, 150 mM NaCl, and 10% glycerol by a homogenizer. The final membrane suspension was stored at −80 °C until use.

### Protein Purification and Crystallization.

The crude membrane suspension was solubilized by adding twice volume of a buffer containing 20 mM Tris-HCl pH 7.5, 150 mM NaCl, 0.02 mg/mL RNAse, 10% (w/v) glycerol, 1% (w/v) *n*-dodecyl-β-D-maltoside (DDM) (Dojindo), and 0.1% (w/v) cholesteryl hemisuccinate (CHS) (Anatrace) with stirring for 1 h. After ultracentrifugation at 200,000×*g* for 1 h, the supernatant was mixed with TALON^®^ cobalt resin (Takara) and stirred for 3 h for binding. The resin was then loaded into a column and washed with a buffer containing 20 mM Tris-HCl pH 7.5, 0.5 M NaCl, 10% (w/v) glycerol, 0.05% DDM, 0.005% CHS, and 15 mM imidazole. Bound proteins were eluted from the resin using the buffer containing 250 mM imidazole. To remove the EGFP tag, the eluates were treated with a 6-His-tagged TEV_SH_ (50) overnight (10:1 mass ratio of HvAACT1 to TEV_SH_). The protein mixture was then precipitated by 30% polyethylene glycol (PEG) 1,500, and the pellet was resuspended in a buffer containing 20 mM Tris-HCl pH 7.5, 0.3 M NaCl, 0.02% DDM, and 0.002% CHS. The protein solution was again loaded onto TALON^®^ resin to remove TEV_SH_ and the uncleaved protein. Finally, the flowthrough was collected and concentrated for size exclusion chromatography (SEC) with a Superdex^®^200 increase 10/300 GL column (Cytiva) in a buffer containing 20 mM Tris-HCl pH 7.5, 0.1 M KCl, and 0.02% DDM. HvAACT1 was concentrated using an Amicon (30 kDa molecular weight cut-off), then flash-frozen by liquid nitrogen and stored at −80 °C until use. Before crystallization, HvAACT1 was precipitated by 30% PEG 400 (Hampton Research) to replace a buffer. After centrifugation of the precipitated HvAACT1 at 17,000×*g* for 20 min, the supernatant was discarded, and HvAACT1 in the pellet was resolubilized into a new buffer containing 20 mM Tris-HCl pH 7.5, 0.1 M KCl, and 0.13% (w/v) Fos-Choline-11 (Anatrace). Crystals were grown using the vapor diffusion sitting-drop method. HvAACT1_cryst_ at 4 mg/mL was mixed (1:1, v/v ratio) with a reservoir solution containing 26 to 30% PEG 300, 0.1 M Na-citrate pH 5.5, 10% (w/v) glycerol, and 2.5% Jeffamine^®^ M-600 (titrated to pH 7, Hampton Research). Crystals usually appeared after 2 d incubating at 8 °C and grew to full size after 2 wk. Crystals were collected and flash-frozen in liquid nitrogen for X-ray diffraction experiments at the synchrotron.

### X-Ray Data Collection, Processing, and Structure Determination.

X-ray diffraction data were collected at the BL41XU beamline, SPring-8 (Japan), and processed with XDS (51) and KAMO (52). With default settings, the XDS output was used for anisotropic truncation and scaling at the Diffraction Anisotropy Server (https://srv.mbi.ucla.edu/Anisoscale/). This resulted in an ellipsoidal truncation with resolutions of 4.4, 4.4, and 3.2 Å along the a*, b*, and c* axes, respectively, and the application of the B-factor. The initial phase information was determined by molecular replacement with Phaser-MR (53), using a polyalanine model of the predicted structure from AlphaFold2 (37). The model was manually built with Coot (54) based on the electron density map calculated with the truncated structural factors from the server and the initial phase. Structural refinement against the original structural factors resulted in very high atomic b-factors (342 Å^2^), and the resultant electron density map was blurred and featureless. On the other hand, the model refinement with the truncated structural factors converged to reasonable b-factors (89 Å^2^) and gave the electron density map with wide side chain features. Therefore, we finally deposited the Protein Data Bank (PDB), the structure refined with the truncated data and two datasets, each consisting of structural factors before and after using the diffraction anisotropy server. The data collection and structural refinement statistics were provided in *SI Appendix*, Table S1. Figures were prepared using CueMol2 (http://www.cuemol.org) or PyMOL (http://www.pymol.org), and the multiple sequence alignment was generated by the clustalW (55), followed by the ESPript 3.0 program. p*K*_a_ values were calculated by H + + server (56).

### Structure Prediction.

The truncated sequence of HvAACT1_cryst_ was provided to AlphaFold2 (37), resulting in five output structures with high predicted Local Distance-Difference Test (pLDDT) (>87) and predicted Template Modeling (>0.87) scores, indicating that they were all reliable. The top four were outward-facing states (#1, #2, #3, and #4), and another was inward-facing (#5). The #1 model was converted to a poly-ALA model and used for the search probe in the molecular replacement of structural determination. The average RMS value of carbon-alpha between the #1 model and the final crystal structure is 0.785 Å^2^, suggesting that the predicted #1 model is similar to the final structure. Notable differences exist in regions with lower pLDDT values, such as the shorter TM2 helix and the longer loop between TM2 and TM3 (L2-3) in the crystal structure. Given the improved, reasonable statistics with the final crystal structure compared to the #1 model, the model bias for using the #1 model for the final crystal structure was negligible.

### MD Simulations.

MD simulations were performed using the AlphaFold2-predicted inward-facing structure of HvAACT1_cryst_, which included residues 57 to 554, with a truncation in the loop region 153 to 199 to match the boundaries of the structure. The initial system was constructed using CHARMM-GUI (57–64), with the transporter embedded in a POPC membrane bilayer in a 150 mM NaCl solution, and trivalent citrate positioned at the putative binding site, which is formed by K126, R358, and R535 (*SI Appendix*, Fig. S14*A*). The system was modeled using the CHARMM36 force field (65, 66). MD simulations were conducted using GROMACS2024 (67, 68). The van der Waals interactions were truncated by applying the force switching function from 10 to 12 Å, while electrostatic interactions were computed using the particle mesh Ewald (69). A holonomic constraint was applied to all bond lengths involving hydrogen atoms using the P-LINCS and SETTLE method (70, 71), with a timestep size of 2 fs for integrating the equations of motion. The velocity rescaling method (72) was used to control the temperature at 310 K, and the cell rescaling method (73) was used to control system pressure at 1 bar. Four different protonation conditions were investigated: The first involved protonating all four potential protonation sites of Asp residues (D92, D99, D289, and D296), the second involved protonating three of them (D92, D289, and D296) or (D99, D289, and D296), and the last involved protonating two of them (D289 and D296). The charged states of other residues were estimated at pH 7. Three independent equilibration simulations were performed for each protonation state with varying initial velocities in the NVT and NPT ensembles for 0.25 ns and 1.3 ns, respectively, following energy minimization by the steepest descent method. Each production MD run was conducted for 500 ns at 310 K and 1 bar under semi-isotropic conditions. To analyze the MD trajectories, we performed the following analyses: First, a time series analysis of the relative distance in the z-coordinate between the center of mass of citrate and the phosphorus atoms of POPC lipids, measured from the membrane center, to observe citrate release events from the protein into the membrane environment, and second, a time series analysis measuring the distances between the center of mass of citrate and the putative binding site residues, K126, R358, and R535.

### Citrate Efflux Activity in Oocytes.

Oocytes for transport activity assay were isolated from *X. laevis*. The ORFs of native and mutated *HvAACT1* were amplified by PCR and inserted into the BglII site of a *Xenopus* oocyte expression vector, pXβG-ev1, with a FLAG tag (DYKDDDDK). Preparation of cRNA, microinjection into oocytes, and efflux transport assay were conducted according to the methods described previously with slight modification (10, 22). A volume of 50 nL cRNA or RNase-free water was injected into the selected oocytes. After 2 d of incubation in Modified Barth’s Saline (MBS) at 18 °C, oocytes with or without HvAACT1/mutated HvAACT1 expression were injected with 50 nL of 2.95 mM ^14^C-labeled citric acid (2.25 nCi/oocytes; PerkinElmer). The oocytes were washed 3 times in low pH MBS (pH 5.0) and then transferred into a new 200 μL buffer at 18 °C. 200 μL of buffer was carefully sampled and replaced with fresh buffer at the indicated time. At the end of the assay, the oocytes were homogenized with 0.1 N HNO_3_. Radioactivity of the external buffer and homogenized oocytes was measured with a liquid scintillation analyzer (Tri-Carb 2800TR; PerkinElmer). Efflux transport activity was presented as % of injected ^14^C radioactivity. Statistical comparison was performed by one-way ANOVA followed by Tukey–Kramer’s test using the software BellCurve for Excel (Social Survey Research Information Co., Ltd). The significance of the differences was defined as *P* < 0.05. All experiments were repeated at least three times independently with consistent results.

## Supplementary Material

Appendix 01 (PDF)

Dataset S01 (XLSX)

## Data Availability

The coordinate and structure factors for HvAACT1_cryst_ have been deposited in PDB with Accession No. 9L7L (74). All other data are included in the manuscript and/or supporting information.
