# Supplementary material for: Structural insights into a citrate transporter that mediates aluminum tolerance in barley
Source: Proc Natl Acad Sci U S A. 2025 Aug 5;122(32):e2501933122. doi: 10.1073/pnas.2501933122 (PMC12358922; doi:10.1073/pnas.2501933122)
Supplement: Supplementary file 1 — Appendix 01 (PDF) [file pnas.2501933122.sapp.pdf]

PNAS

Supporting Information for
Structural insights into a citrate transporter that mediates aluminum tolerance in barley

Tran Nguyen Thao[a], Namiki Mitani-Ueno[b], Ryo Urano[c], Yasunori Saitoh[a, c], Peitong Wang[b, d], Naoki Yamaji[b], Jian-Ren Shen[a, c], Wataru Shinoda[a, c], Jian Feng Ma[b, 1], Michihiro Suga[a, c, 1]

[a]Graduate School of Environmental, Life, Natural Science, and Technology, Okayama University, Okayama, 700-8530, Japan.
[b]Institute of Plant Science and Resources, Okayama University, Kurashiki, 710-0046, Japan
[c]Research Institute for Interdisciplinary Science, Okayama University, Okayama, 700-8530, Japan
[d]National Key Laboratory of Crop Genetics & Germplasm Enhancement and Utilization, College of Resources and Environmental Sciences, Nanjing Agricultural University, Nanjing 210095, China

[1]Correspondence: Jian Feng Ma, Michihiro Suga
Email:maj@okayama-u.ac.jp or michisuga@okayama-u.ac.jp

This PDF file includes:

Supporting text
Figures S1 to S19
Tables S1 to S4

Other supporting materials for this manuscript include the following:

Datasets S1

Supporting Information Text

Effects of Protonation states on Citrate binding stability

Molecular dynamics (MD) simulations demonstrated that the key Asp residues' protonation state significantly influenced the citrate binding's stability. When all four Asp residues ($D92^{TM1}$, $D99^{TM1}$, $D289^{TM5}$, and $D296^{TM5}$) were protonated, citrate remained bound to the transporter throughout the 500 ns simulation in all three replicates (*SI Appendix*, Fig. S15). Citrate remained tightly bound to $K126^{TM2}$ for 500 ns (*SI Appendix*, Fig. S15A). In contrast, the distances between citrate and residues $R358^{TM7}$ and $R535^{TM12}$ exceeded 10 Å, indicating minimal interaction with them (*SI Appendix*, Fig. S15B and C). Instead, citrate primarily occupied the $K126^{TM2}$+$R255^{TM4}$ and $K126^{TM2}$+$R273^{TM4}$ binding states. *SI Appendix*, Fig. S15D demonstrates that citrate remained within the transporter throughout all three simulations. Under these conditions, citrate mainly interacted with $K126^{TM2}$, $R255^{TM4}$, and $R273^{TM4}$, while interactions with $R358^{TM7}$ and $R535^{TM12}$ were rarely observed.

When three aspartate residues ($D92^{TM1}$, $D289^{TM5}$, and $D296^{TM5}$) were protonated, the interaction with $K126^{TM2}$ was maintained throughout the 500 ns period, with citrate transitioning between three binding modes: $K126^{TM2}$+$R255^{TM4}$, $K126^{TM2}$+$R273^{TM4}$, and $K126^{TM2}$+$R358^{TM7}$+$R535^{TM12}$, as illustrated in *SI Appendix*, Fig. S16. The oscillating distances between 0-10 Å indicate that citrate alternated between the $K126^{TM2}$+$R358^{TM7}$+$R535^{TM12}$ binding site and other sites, suggesting dynamic movement of citrate within the binding pocket throughout the simulation period (*SI Appendix*, Fig. S16). When three aspartate residues ($D99^{TM1}$, $D289^{TM5}$, and $D296^{TM5}$) were protonated, citrate binding behavior varied among the replicates. In two replicates, the interaction with $K126^{TM2}$ was maintained throughout the 500 ns period, similar to when three aspartate residues ($D92^{TM1}$, $D289^{TM5}$, and $D296^{TM5}$) were protonated. However, in one simulation, the interaction with $K126^{TM2}$ was lost after approximately 380 ns, leading to the release of citrate from the transporter (*SI Appendix*, Fig. S17).

When only two aspartate residues ($D289^{TM5}$ and $D296^{TM5}$) were protonated, citrate was released from the transporter into the membrane environment within tens of nanoseconds in two of the three replicates (*SI Appendix*, Fig. S18D). In the remaining simulation, citrate continued to transition between the three binding sites; however, it spent more time in the $K126^{TM2}$+$R358^{TM7}$+$R535^{TM12}$ binding site compared to the three-protonation state (*SI Appendix*, Fig. S18A-C).

Transition of Citrate to the Binding Sites

A key finding from the MD simulations was the relationship between the protonation state and citrate positioning within the binding site. As the number of protonated aspartate residues decreased from four to two, citrate exhibited weaker binding to the transporter and an increased probability of transitioning from the $R255^{TM4}$ or $R273^{TM4}$ sites to the $R358^{TM7}$+$R535^{TM12}$ site. This positional shift may reflect the transport mechanism, suggesting that changes in protonation state drive the movement of citrate through the transport pathway.

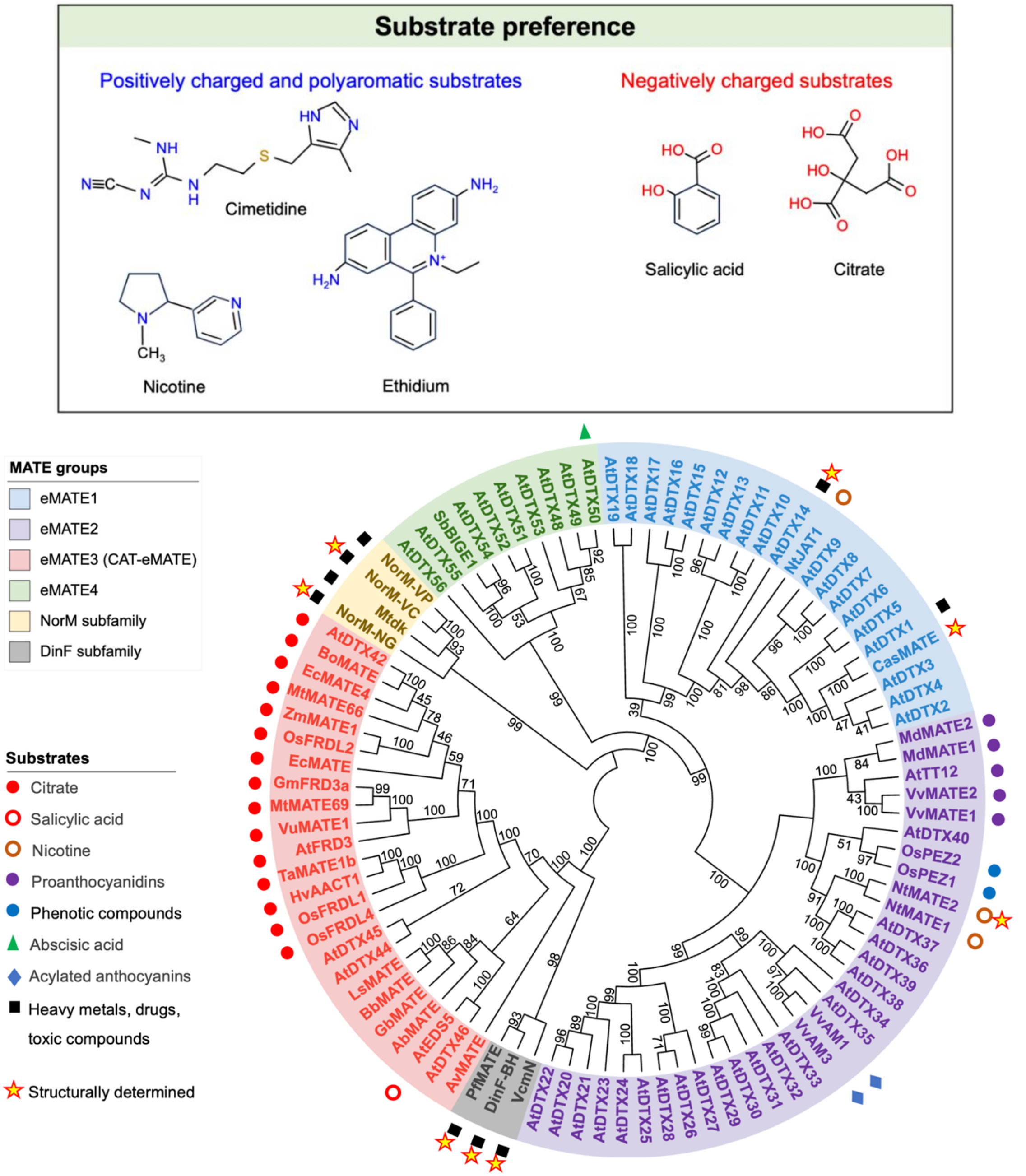

Fig. S1. Phylogenetic tree of Multidrug and toxic compound extrusion (MATE) transporters and their substrate preferences.

Protein sequence IDs were listed in Table S4. Proteins with published structures are marked with a star. Substrate: citrate (red filled circles); salicylic acid (red open circle); nicotine (brown open circle); proanthocyanidins (purple circle), phenolic compounds (blue circle); abscisic acid (green triangle); heavy metals, drugs, and toxic compounds (black square); acylated anthocyanins (blue diamond). The alignment was constructed with clustalW and the phylogenetic tree was constructed with MEGA 12. The bootstrap values were presented in each branch.

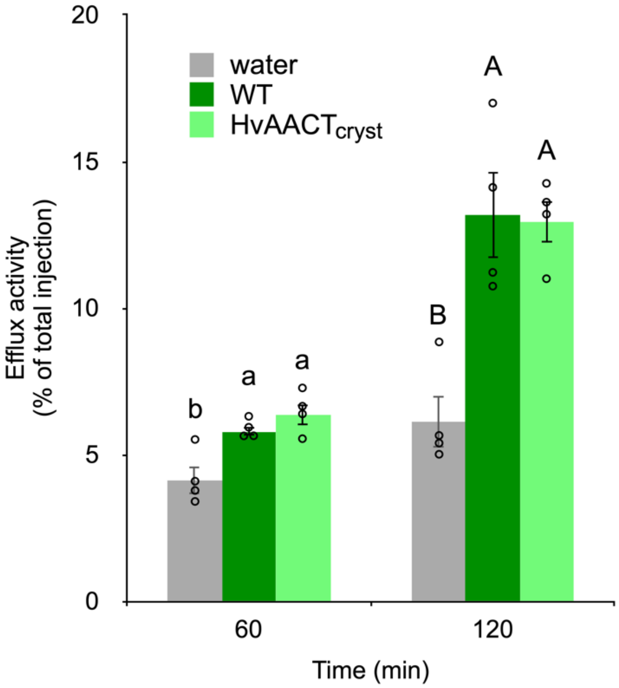

Fig. S2. Citrate efflux activity of HvAACT1 wild type (WT) and HvAACT1$_{cryst}$ expressed in *Xenopus* oocytes.

Water or cRNA of wild-type HvAACT1 or HvAACT1$_{cryst}$ were injected into oocytes. After 2 days of cultivation, the oocytes were injected with 2.95 mM ^{14}C-labeled citrate. The release of ^{14}C-labeled citrate from the oocytes was determined at various times. Data are given as means ± SEM (*n*=4, each replicate contains 5 oocytes). Different letters indicate significant differences with $p < 0.05$ by Tukey-Kramer's test.

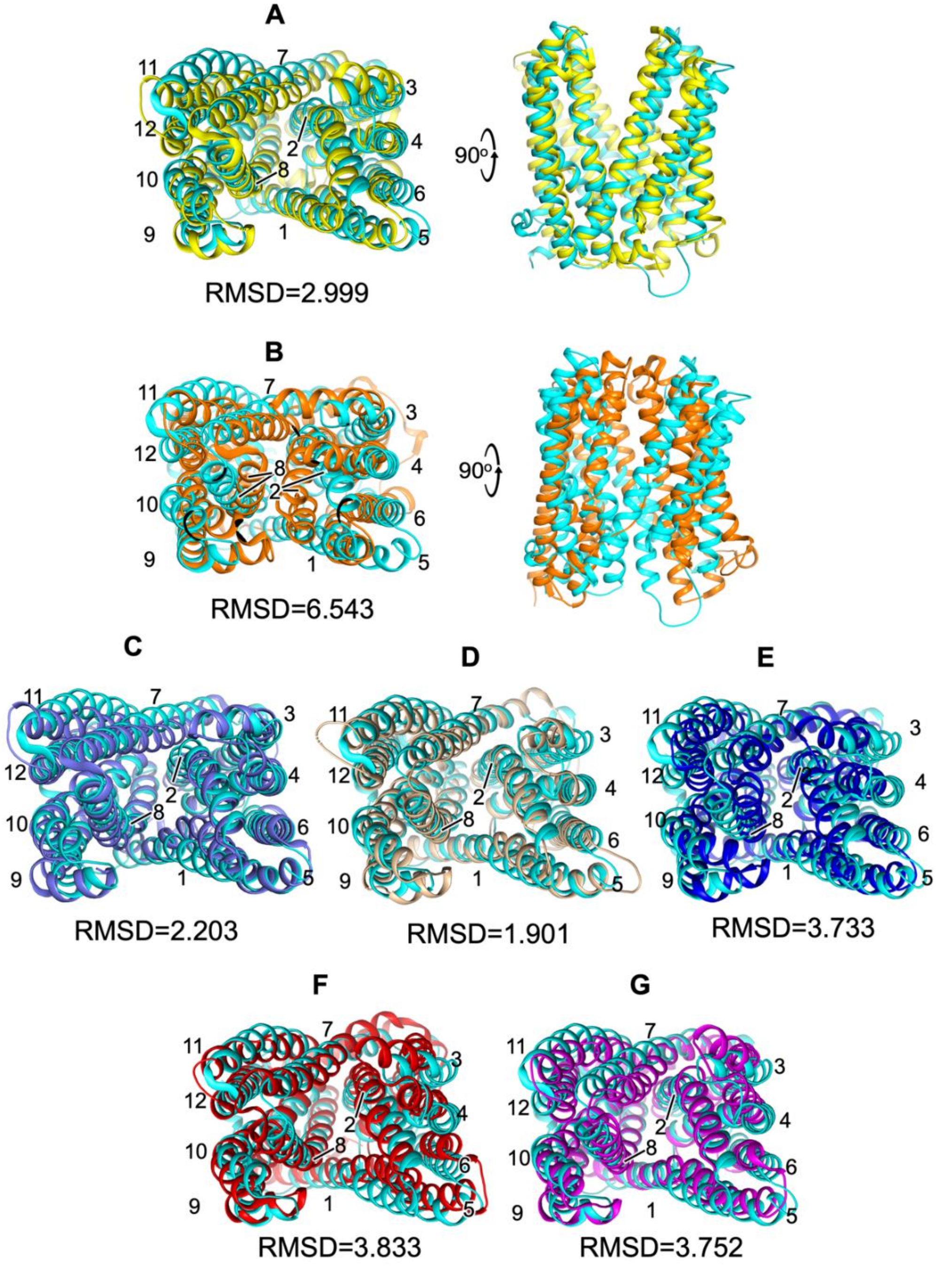

Fig. S3. Structural comparison between HvAACT1$_{cryst}$ (cyan) and other published structures of MATE transporters

Superposition of HvAACT1 with **(A)** outward-facing PfMATE TM1-straight (PDB 3VVN, yellow), **(B)** inward-facing PfMATE (PDB 6FHZ, orange), **(C)** outward-facing PfMATE TM1-bent (PDB 3VVO, light blue), **(D)** NorM-VC (PDB 7PHP, wheat), **(E)** AtDTX14 (PDB 5Y50, blue), **(F)** NtMATE2 (PDB 7DQK, red), and **(G)** CasMATE (PDB 5YCK, magenta), viewed from the apoplast **(A** to **G)** and the plane of the membrane **(A** and **B)** are shown. Numbers represent transmembrane helices. The RMSD value (Å) between each pair is specified at the bottom.

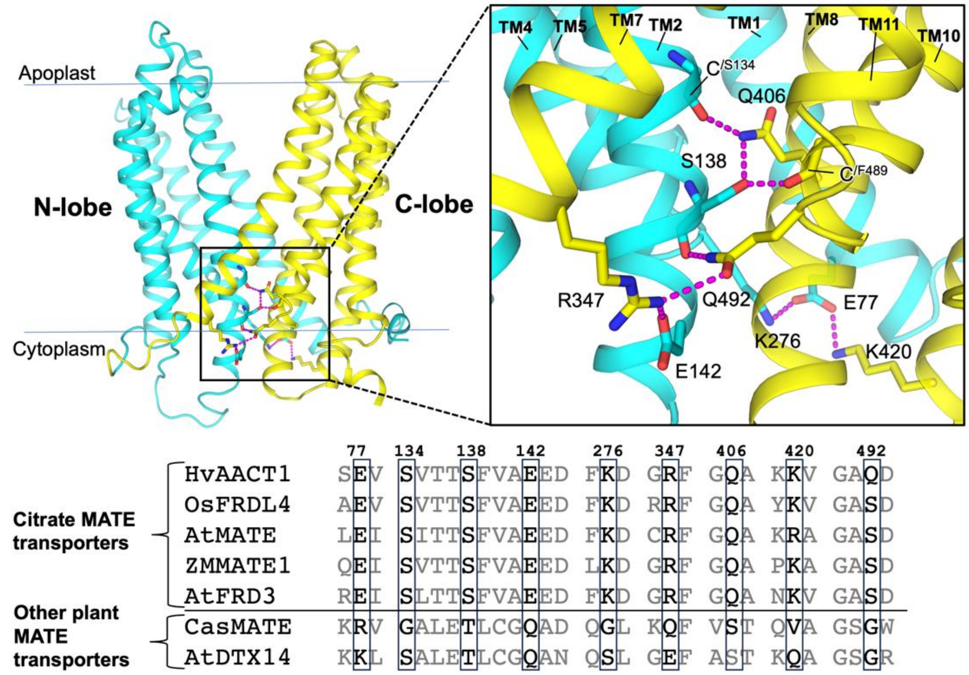

Fig. S4. An intercellular gate of the outward-facing HvAACT1$_{cryst}$ structure.

Key interactions between the N-lobe (cyan) and C-lobe (yellow) interface are represented by magenta dashes. Their partial sequence alignment of plant MATE transporters is also presented.

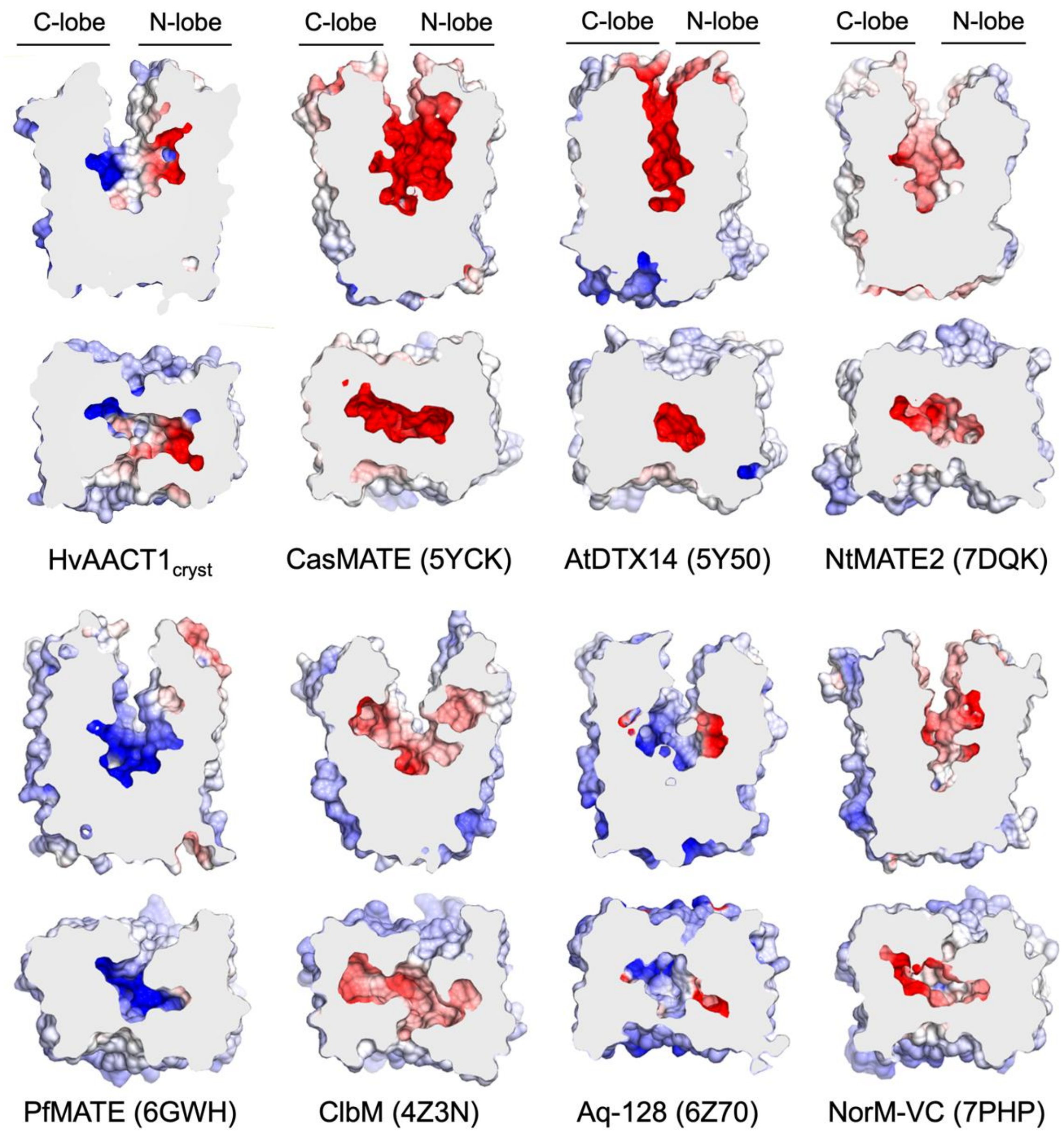

Fig. S5. Electrostatic surface potential maps of the cavity in HvAACT1$_{cryst}$ and other published MATE transporters.

The electrostatic surface potential (red, anionic; blue, cationic; white, uncharged) was calculated the same way as Fig. 2B, with the view directions also being the same. The N-lobe is on the right half, and the C-lobe is on the left half of each structure.

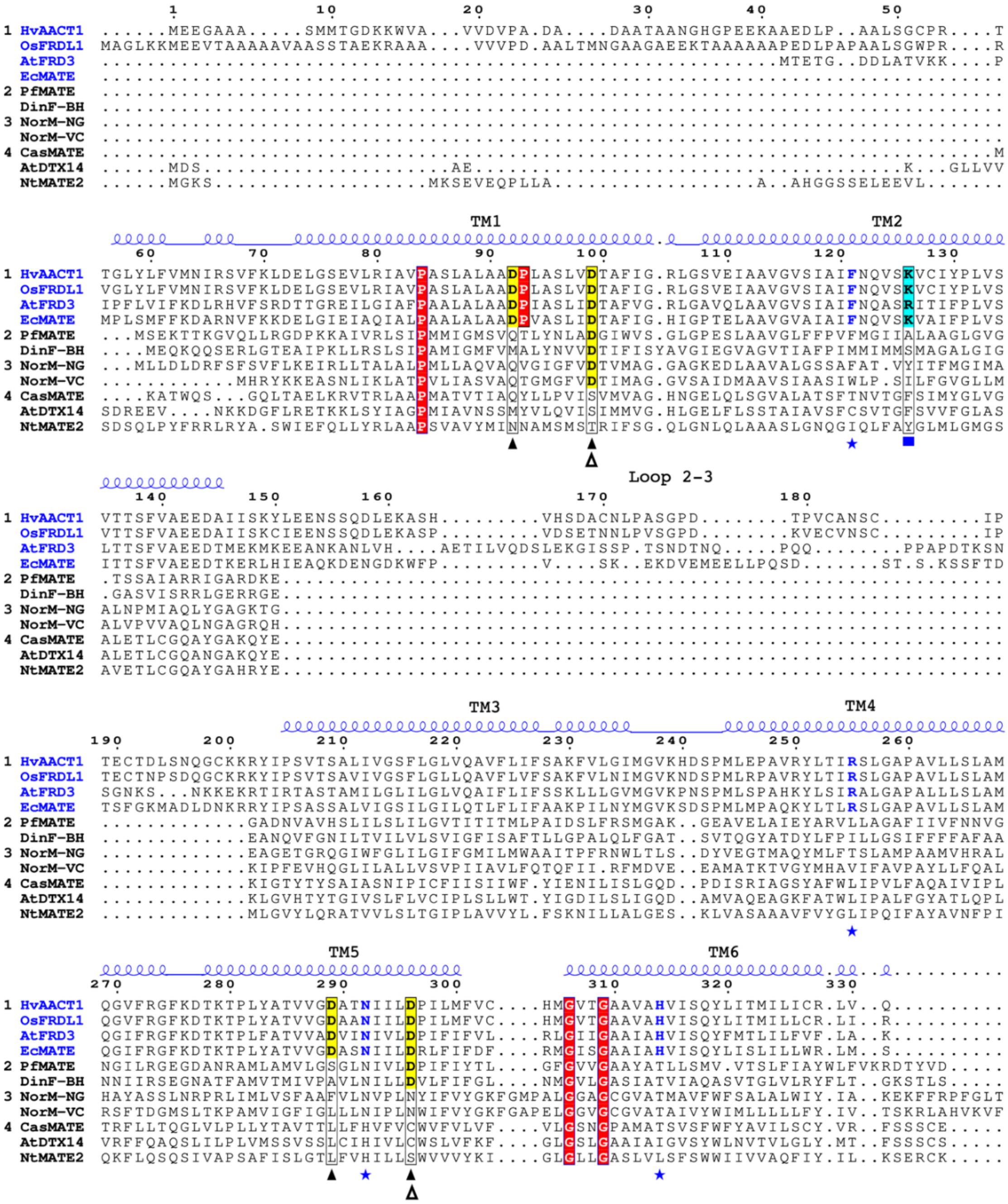
1 HvAACT1 ......MEEGAAA.....SMMTGDKKWVA...VVDVPA.DA....DAATAANGHGPEEKAAEDLP..AALSGCPR....T
OsFRDL1 MAGLKKMEEVTAAAAAVAASSTAEKRAAA....VVVPD.AALTMNGAAGAEEKTAAAAAAPEDLPAPAALSGWPR....R
AtFRD3 ....MTETG..DDLATVKK....P
EcMATE
2 PfMATE
DinF-BH
3 NorM-NG
NorM-VC
4 CasMATE ....M
AtDTX14 ......MDS...................AE..............................K...GLLVV
NtMATE2 ......MGKS.................MKSEVEQPLLA..................A..AHGGSSELEEVL.......
TM1
TM2
1 HvAACT1 TGLYLFVMNIRSVFKLDELGSEVLRIAVPASLALAADPLASLVDTAFIG.RLGSVEIAAVGVSIAIFNQVSKVCIYPLVS
OsFRDL1 VGLYLFVMNIRSVFKLDELGSEVLRIAVPASLALAADPLASLVDTAFIG.RLGSVEIAAVGVSIAIFNQVSKVCIYPLVS
AtFRD3 IPFLVIFKDLRHVFSRDTTGREILGIAFPAALALAADPIASLIDTAFVG.RLGAVQLAAVGVSIAIFNQASRITIFPLVS
EcMATE MPLSMFFKDARNVFKKDELGIEIAQIALPAALALAADPVASLIDTAFIG.HIGPTELAAVGVAIAIFNQVSKVAIFPLVS
2 PfMATE ...MSEKTTKGVQLLRGDPKKAIVRLSIPMMIGMSVQTLYNLADGIWVS.GLGPESLAAVGLFFPVFMGIIALAAGLGVG
DinF-BH ....MEQKQQSERLGTEAIPKLLRSLSIPAMIGMFVMALYNVVDTIFISYAVGIEGVAGVTIAFPIMMIMMSMAGALGIG
3 NorM-NG ...MLLDLDRFSFSVFLKEIRLLTALALPMLLAQVAQVGIGFVDTVMAG.GAGKEDLAAVALGSSAFAT.VYITFMGIMA
NorM-VC ...........MHRYKKEASNLIKLATPVLIASVAQTGMGFVDTIMAG.GVSAIDMAAVSIAASIWLP.SILFGVGLLM
4 CasMATE ....KATWQS...GQLTAELKRVTRLAAPMATVTIAQYLLPVISVMVAG.HNGELQLSGVALATSFTNVTGFSIMYGLVG
AtDTX14 SDREEV....NKKDGFLRETKKLSYIAGPMIAVNSSMYVLQVISIMMVG.HLGELFLSSTAIAVSFCSVTGFSVVFGLAS
NtMATE2 SDSQLPYFRRLRYA.SWIEFQLLYRLAAPSVAVYMINNAMSMSTRIFSG.QLGNLQLAAASLGNQGIQLFAYGLMLGMGS
Loop 2-3
1 HvAACT1 VTTSFVAEEDAIISKYLEENSSQDLEKASH.........VHSDACNLPASGPD........TPVCANSC.........IP
OsFRDL1 VTTSFVAEEDAIISKCIEENSSQDLEKASP.........VDSETNNLPVSGPD........KVECVNSC.........IP
AtFRD3 LTTSFVAEEDTMEKMKEEANKANLVH....AETILVQDSLEKGISSP.TSNDTNQ.....PQQ........PPAPDTKSN
EcMATE ITTSFVAEEDTKERLHIEAQKDENGDKWFP.........V....SK..EKDVEMEELLPQSD.......ST.S.KSSFTD
2 PfMATE .TSSAIARRIGARDKE
DinF-BH .GASVISRRLGERRGE
3 NorM-NG ALNPMIAQLYGAGKTG
NorM-VC ALVPVVAQLNGAGRQH
4 CasMATE ALETLCGQAYGAKQYE
AtDTX14 ALETLCGQANGAKQYE
NtMATE2 AVETLCGQAYGAHRYE
TM3
TM4
1 HvAACT1 TECTDLSNQGCKKRYIPSVTSALIVGSFLGLVQAVFLIFSAKFVLGIMGVKHDSPMLEPAVRYLTIRSLGAPAVLLSLAM
OsFRDL1 TECTNPSDQGCKRKYIPSVTSAVIVGSFLGLLQAVFLVFSAKFVLNIMGVKNDSPMLRPAVRYLTIRSLGAPAVLLSLAM
AtFRD3 SGNKS...NKKEKRTIRTASTAMILGLILGLVQAIFLIFSSKLLLGVMGVKPNSPMLSPAHKYLSIRALGAPALLLSLAM
EcMATE TSFGKMADLDNKRRYIPSASSALVIGSILGILQTLFLIFAAKPILNYMGVKSDSPMLMPAQKYLTLRSLGAPAVLLSLAM
2 PfMATE ............GADNVAVHSLILSLILGVTITITMLPAIDSLFRSMGAK..GEAVELAIEYARVLLAGAFIIVFNNVG
DinF-BH ............EANQVFGNILTVILVLSVIGFISAFTLLGPALQLFGAT..SVTQGYATDYLFPILLGSIFFFFAFAA
3 NorM-NG ............EAGETGRQGIWFGLILGIFGMILMWAAITPFRNWLTLS..DYVEGTMAQYMLFTSLAMPAAMVHRAL
NorM-VC ............KIPFEVHQGLILALLVSVPIIAVLFQTQFII.RFMDVE..EAMATKTVGYMHAVIFAVPAYLLFQAL
4 CasMATE ............KIGTYTYSAIASNIPICFIISIIWF.YIENILISLGQD..PDISRIAGSYAFWLIPVLFAQAIVIPL
AtDTX14 ............KLGVHTYTGIVSLFLVCIPLSLLWT.YIGDILSLIGQD..AMVAQEAGKFATWLIPALFGYATLQPL
NtMATE2 ............MLGVYLQRATVVLSLTGIPLAVVYL.FSKNILLALGES..KLVASAAAVFVYGLIPQIFAYAVNFPI
TM5
TM6
1 HvAACT1 QGVFRGFKDTKTPLYATVVGDATNIILDPILMFVC....HMGVTGAAVAHVISQYLITMILICR.LV..Q.........
OsFRDL1 QGVFRGFKDTKTPLYATVVGDAANIILDPILMFVC....HMGVTGAAVAHVISQYLITMILLCR.LI..R.........
AtFRD3 QGIFRGFKDTKTPLFATVVADVINIVLDPIFIFVL....RLGIIGAAIAHVISQYFMTLILFVF.LA..K.........
EcMATE QGIFRGFKDTKTPLYATVVGDASNIILDRLFIFDF....RMGISGAAIAHVISQYLISLILLWR.LM..S.........
2 PfMATE NGILRGEGDANRAMLAMVLGSGLNIVLDPIFIYTL....GFGVVGAAYATLLSMV.VTSLFIAYWLFVKRDTYVD.....
DinF-BH NNIIRSEGNATFAMVTMIVPAVLNILLDVLFIFGL....NMGVLGASIATVIAQASVTGLVLRYFLT..GKSTLS.....
3 NorM-NG HAYASSLNRPRLIMLVSFAAFVLNVPLNYIFVYGKFGMPALGGAGCGVATMAVFWFSALALWIY.IA..KEKFFRPFGLT
NorM-VC RSFTDGMSLTKPAMVIGFIGLLLNIPLNWIFVYGKFGAPELGGVGCGVATAIVYWIMLLLLLFY.IV..TSKRLAHVKVF
4 CasMATE TRFLLTQGLVLPLLYTAVTTLLFHVFVCWVFVLVF....VLGSNGPAMATSVSFWFYAVILSCY.VR..FSSSCE.....
AtDTX14 VRFFQAQSLILPLVMSSVSSLCIHIVLCWSLVFKF....GLGSLGAAIAIGVSYWLNVTVLGLY.MT..FSSSCS.....
NtMATE2 QKFLQSQSIVAPSAFISLGTLFVHILLSWVVVYKI....GLGLLGASLVLSFSWWIIVVAQFIY.IL..KSERCK.....

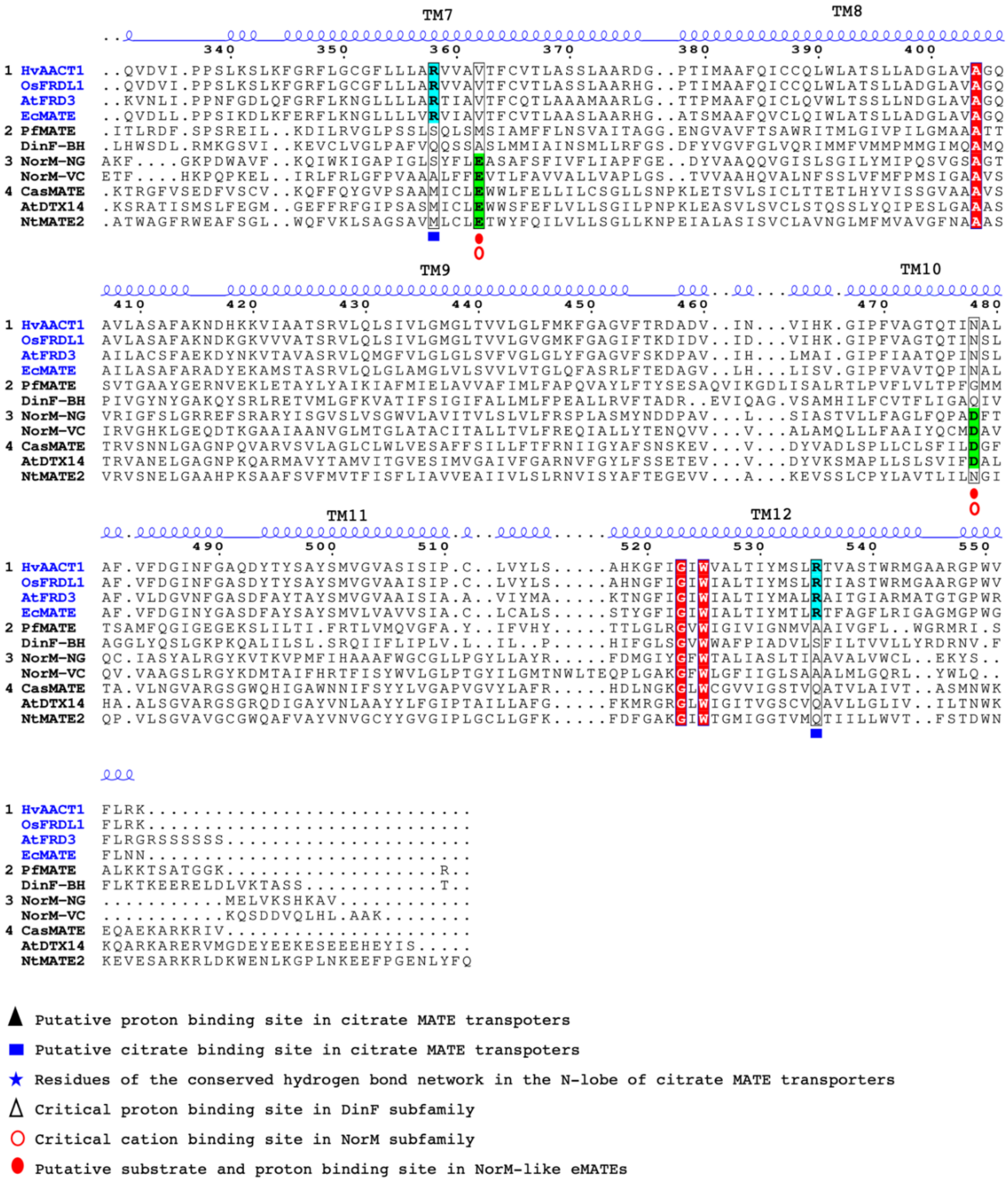

Fig. S6. Sequence alignment of MATE transporters.

Group 1, citrate MATE transporters; group 2, DinF subfamily; 3, NorM subfamily; group 4, other plant MATE transporters of the eMATE subfamily. The secondary structure of $HvAACT1_{cryst}$ is labeled on top with the amino acid number in HvAACT1. Residues strictly conserved across all three subfamilies are highlighted in red, and those critical for MATE transporters' functionality are marked as explained at the bottom panel.

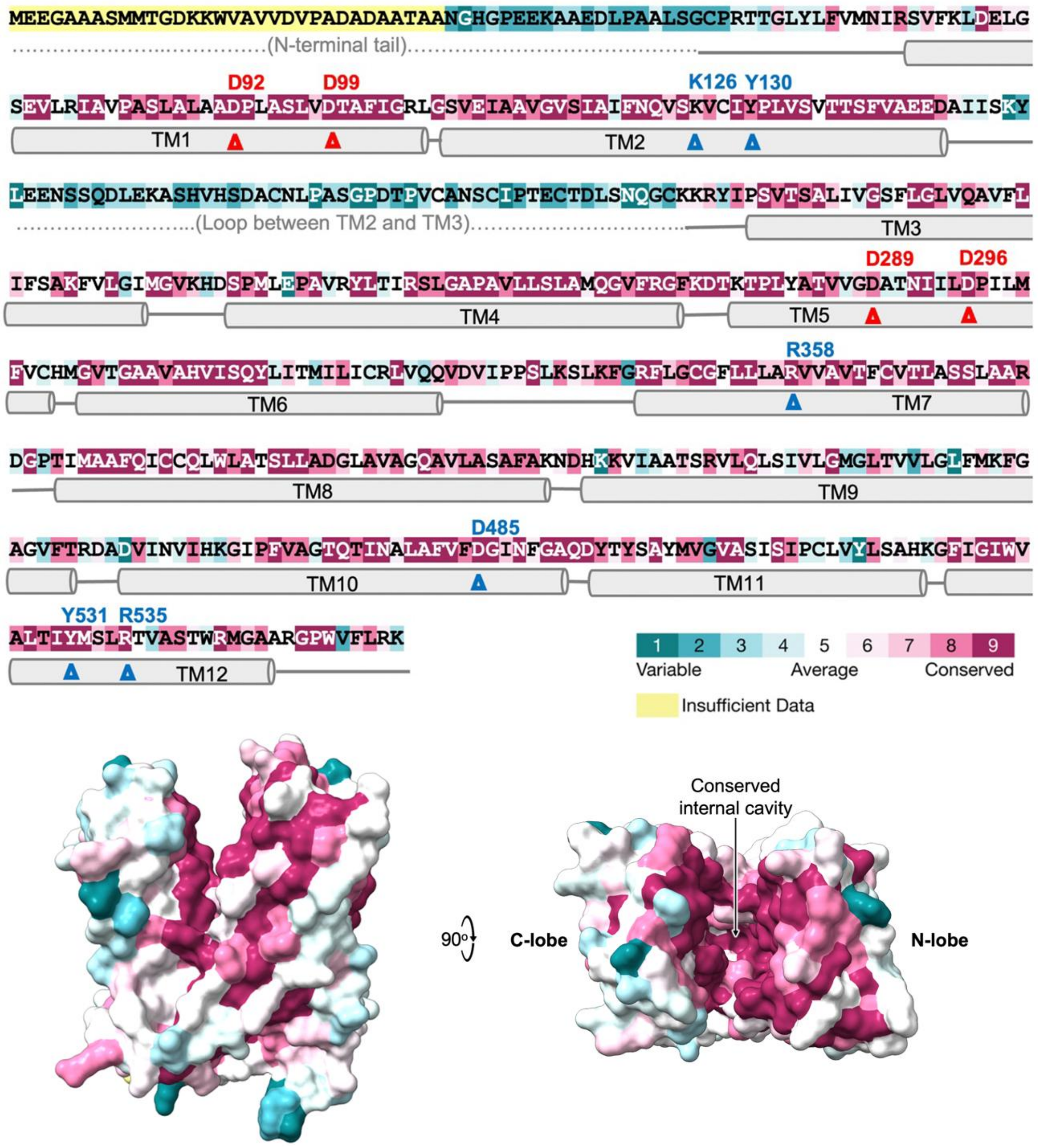

Fig. S7. Sequence conservation of HvAACT1 among citrate MATE transporters.

Protein sequences of fifteen citrate MATE transporters with HvAACT1 have been aligned. The Consurf Server then analyzed the result to score each residue from 1 (most variable) to 9 (most conserved). ChimeraX visualized the surface of HvAACT1$_{cryst}$ to show the conserved internal cavity of the protein. Amino acids marked with red triangles (Δ) are essential for cation/H^+ binding in the N-lobe, while those marked with blue triangles (Δ) take part in the hydrogen bond network within the C-lobe, which is important for substrate binding. Cylinders represent transmembrane helices of HvAACT1$_{cryst}$.

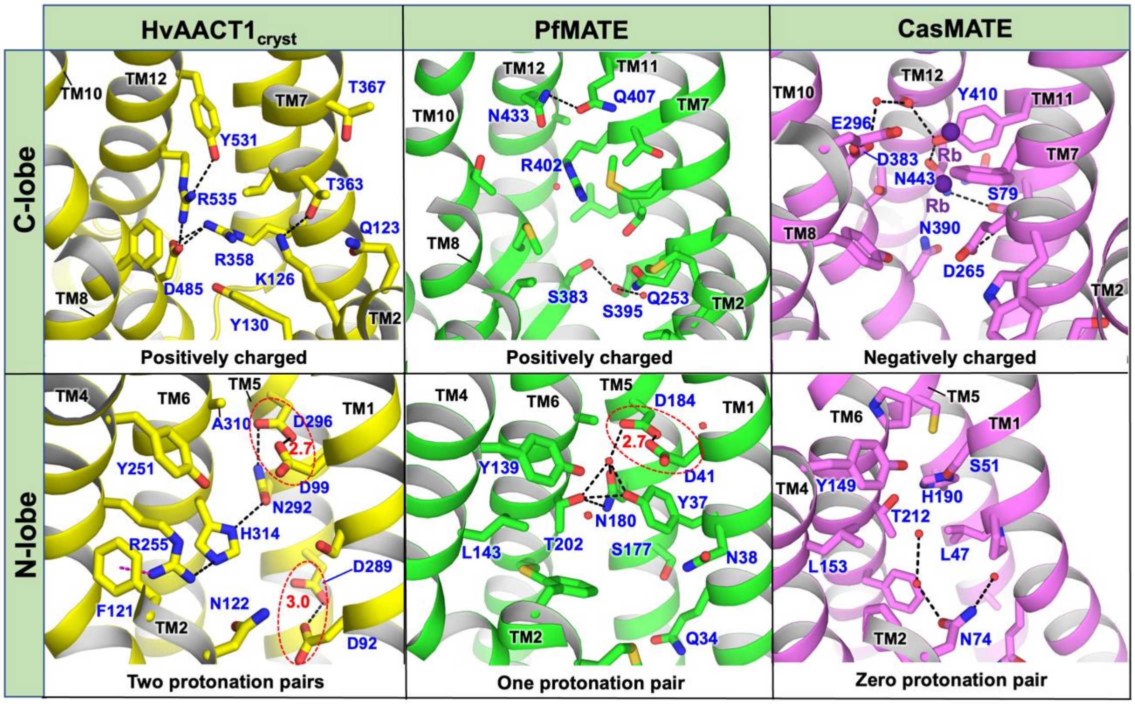


```
               84    92 93        99   126   289        296   358                 535
HvAACT1     VPA AADPLASLVDT SKV GDATNIILDP ARVVAVT TINAL SLRTV
AtFRD3      FPA TADPLASLIDT SRI ADVINIVLDP ARTIAVT PINSL ALRAI
PfMATE      IPM SVQTLYNLADG IAL GSGLNIVLDP LSQLSMS VLTPF NMVAA
NorM-NG     LPM VAQVGIGFVDT YIT AFVLNVPLNY LSYFLEA PADFT TIAAV
NtMATE2     APS MINNAMSMSTR YGL TLFVHILLSW VMLCLET ILNGI VMQTI
CasMATE     APM IAQYLLPVISV FSI TLLFHVFVCW AMICLEW ILDGF TVQAT
AtDTX14     GPM SSMYVLQVISI FSV SLCIHIVLCW SMICLEW IFDAL CVQAV
                                                   265    383
```

Fig. S8. Structural comparisons at N-lobes and C-lobes.

The C-lobes and N-lobes of $HvAACT1_{cryst}$ (yellow), PfMATE (green), and CasMATE (magenta) were shown with their crucial residues in stick representation. Dashed black lines indicate hydrogen bonds; magenta dashed lines indicate cation-pi interaction; and red spheres are water molecules. The lowest panel presents the partial alignment of MATE transporters from different subfamilies. The substrate or counter ion transport residues were colored blue and red, respectively. Proline residues that are critical for TM1 bending are colored green. Numbers above and below the alignment correspond to residual numbers in HvAACT1 and AtDTX14, respectively.

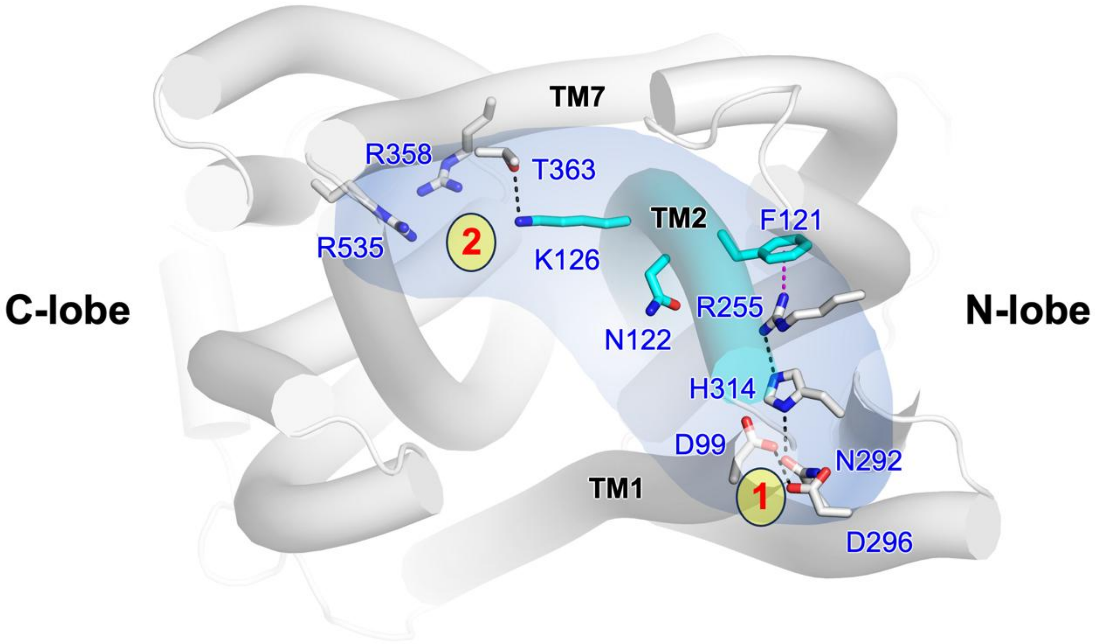

Fig. S9. The Hydrogen bond network connects the N- and C-lobes.

The hydrogen-bond network that connects the N-lobe protonation site (marked as 1) and the C-lobe substrate binding site (marked as 2) via TM2. Hydrogen bonds and the cation-π interaction in the network are shown as black and magenta dashed lines, respectively.

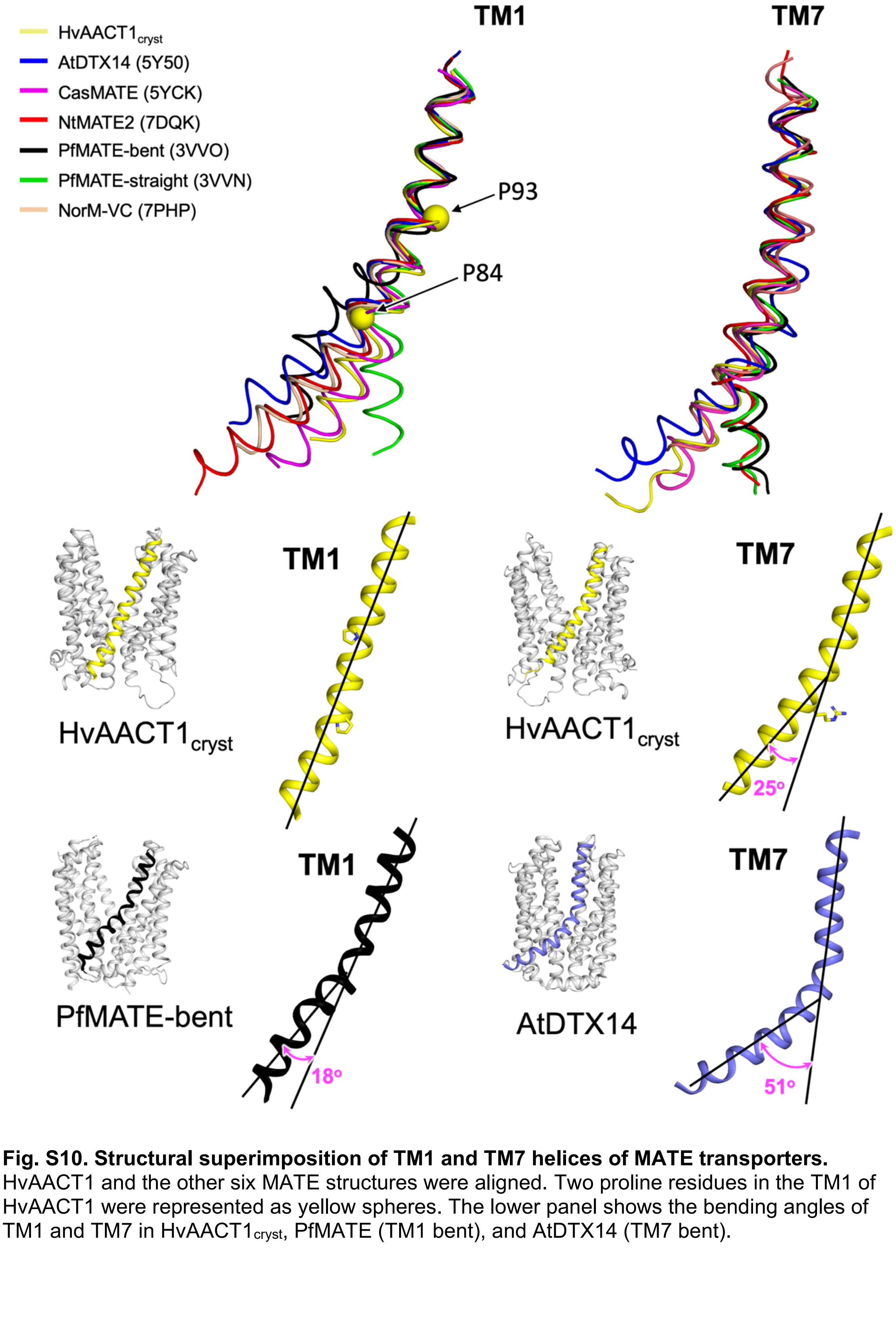

Fig. S10. Structural superimposition of TM1 and TM7 helices of MATE transporters. HvAACT1 and the other six MATE structures were aligned. Two proline residues in the TM1 of HvAACT1 were represented as yellow spheres. The lower panel shows the bending angles of TM1 and TM7 in HvAACT1$_{cryst}$, PfMATE (TM1 bent), and AtDTX14 (TM7 bent).

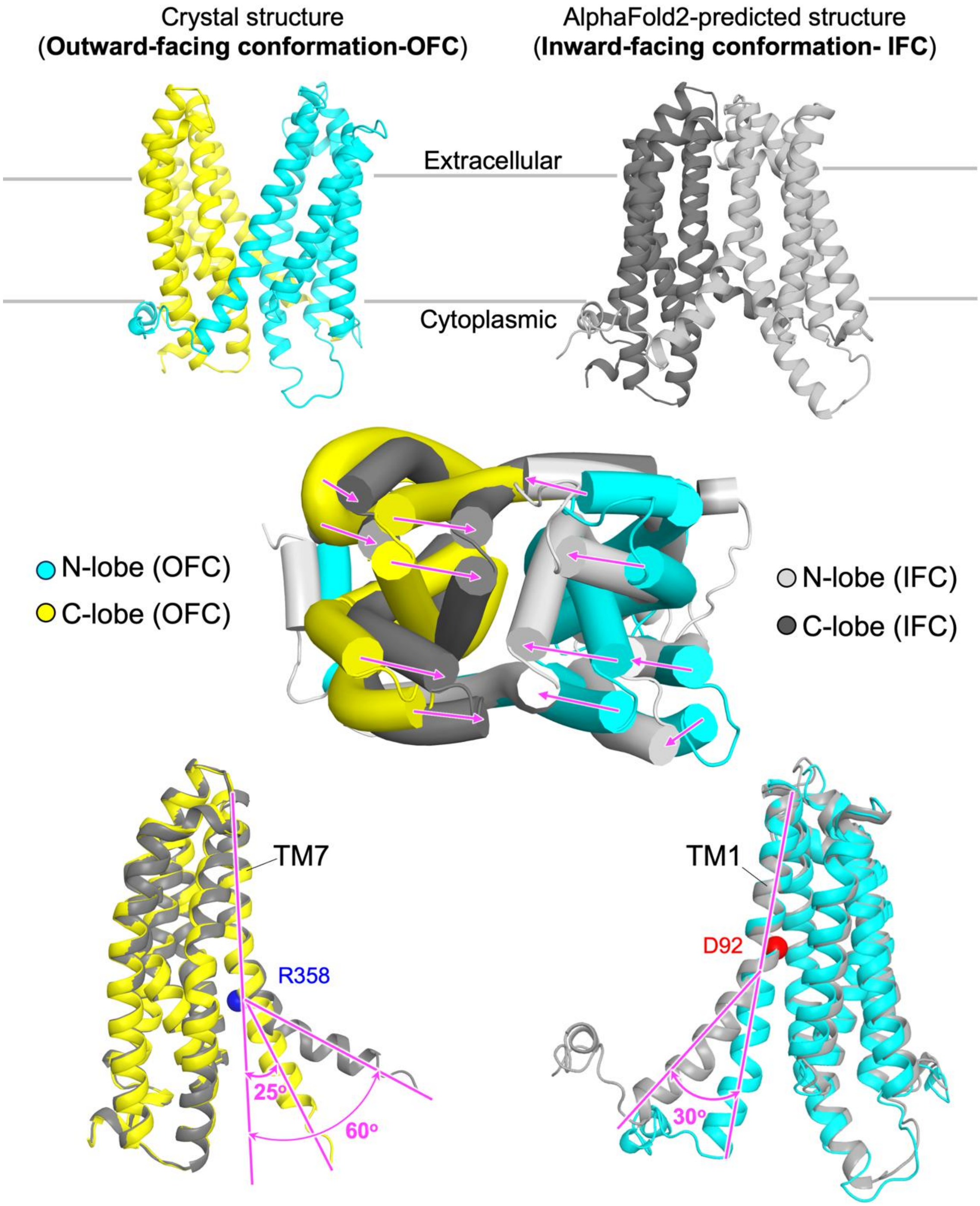

Fig. S11. Structural comparisons between the outward-facing structure and the inward-facing AlphaFold2-predicted structure of HvAACT1$_{cryst}$.

The outward and inward-facing structures are shown in color and gray, respectively. The N-lobe and C-lobe of these structures were aligned separately to prepare the superposed structures. The bending angles of TM1 and TM7 in each structure were estimated.

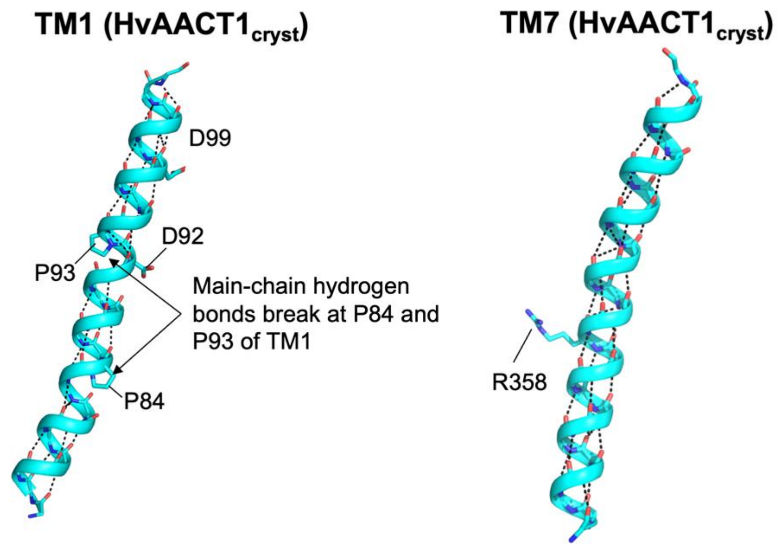

Fig. S12. TM1 and TM7 in the outward-facing structure of HvAACT1$_{cryst}$.

Key residues in TM1 (P84, P93, D92, and D99) and TM7 (R358) are highlighted in stick presentation.

```
                             1        10        20        30        40
HvAACT1     .................MEEG..AAASMMTGDKKWVAVVDVPADADAATAANGHGPEEK......AAED.....LPA..A
AtDTX42     ...............................................................MMSEDG.......YN..
BoMATE      ...............................................................MMSEDG.......YTKE
MtMATE66    ...............................................................MAEKES.......LFS.
EcMATE4     .....................................................MEPLEGSIMGTTSSEE.......DYPF
EcMATE      ................................................................................
ZmMATE1     ...............MHPPTHMRARAPIGNSGPDSLSYFIPLHPLLGVWTMHGGRERERESLLFLPDPS.......AGAM
OsFRDL2     ..................................................MNGESLLDRSSS...ADAA.......MNDA
MtMATE69    ................................................................MDDNDI.......SNN
VuMATE1     ................................................................MEENG........SSN
AtFRD3      ................................................................MTETG.........DD
TaMATE1b    .................MEEG..AAASMTVGEK..RVAVDVPAG..AAAAANGHGPEEK......AAED.....VPAPSA
OsFRDL1     ...........MAGLKKMEEVTAAAAAVAASSTAEKRAAAVVVPDAALTMNGAAGAEEKTAAAAAAPED.....LPAPAA
OsFRDL4     .......MARSSSAPESMEADHQLTATVTAAASGDMPVTEQQQQKQCMVAVAAPPATENADAAGGGGGDNGDHLPPTATS
AtDTX45     MESSRVVVGGGLPLANRRNSS.FAKPKIQQGTFLPLSRINNVSAPQKCSLHTNPNPMFPFVTRRKSQTNPDCGVVKLGEE
AtDTX44     ....MAAVATSFCFSPHRSPSRFGNP..............NSSIRRTIVCKSSPRDESPAVS..TSSQRPE.......KQ
LsMATE      ................................................................................
BbMATE      ................................................................................
GbMATE      ................................................................................
AbMATE      ................................................................................
AtEDS5      .......MLIKSQRLTLFSPLLSKTRRIPVNSHQTLVAES.VITRRTLGAITATPS......FHKNPVVIRRRIKLERVT
AtDTX46     .......MQIQCKTLTFTVSSIPCNPKLPFPSSLTLRSWNPSFPSFRSSAVSGPKSSLKLNRFLRNCASTNQELVVDGET
AvMATE      ................................................................................
PfMATE      ................................................................................
DinF-BH     ................................................................................
VcmN        ................................................................................
NorM-VP     ................................................................................
NorM-VC     ................................................................................
Mtdk        ................................................................................
NorM-NG     ................................................................................
AtDTX19     ................................................................................
AtDTX18     ................................................................................
AtDTX17     ................................................................................
AtDTX16     ................................................................................
AtDTX15     ................................................................................
AtDTX12     ................................................................................
AtDTX13     ................................................................................
AtDTX11     ................................................................................
AtDTX10     ................................................................................
AtDTX14     ................................................................................
NtJAT1      ................................................................................
AtDTX9      ............................................................................MKKS
AtDTX8      ................................................................................
AtDTX7      ................................................................................
AtDTX6      ................................................................................
AtDTX5      ................................................................................
AtDTX1      ................................................................................
CasMATE     ................................................................................
AtDTX3      ................................................................................
AtDTX4      ................................................................................
AtDTX2      ................................................................................
MdMATE2     .....................................................MGTAEEYQ.................PL
MdMATE1     .....................................................MGSQEEYQ.................PL
AtTT12      .....................................................MSSTETYE.................PL
VvMATE2     ......................................................MGSEEYQ.................PL
VvMATE1     ....................................................MASAAEDGE.................PL
AtDTX40     .....................................................MDSSPNDG...............VHQP
OsPEZ2      ................................................................................
OsPEZ1      .....................................................MGSSVKDAGGGKEEQQQQLESPLLEAA
NtMATE2     .....................................................MGKS.....................MK
NtMATE1     .....................................................MGKS.....................MK
AtDTX37     .....................................................MN..SES...................L
AtDTX36     .....................................................MG..SEAT................TAV
AtDTX39     .....................................................MDVSNETV................ER.
AtDTX38     .....................................................MNGSNETV................ERR
AtDTX34     .................................MEIPVREERRSSSSSAGPLQQTISLAADDAIDSGPSSPLVVKVSVFE
AtDTX35     ................................................................................
VvAM3       ................................................................................
VvAM1       ................................................................................
AtDTX33     ............................................................................MGKD
AtDTX32     ............................................................................METL
AtDTX31     ............................................................................MEKD
AtDTX30     ............................................................................MEED
AtDTX29     ............................................................................MAKD
AtDTX27     ................................................................................
AtDTX26     ................................................................................
AtDTX28     ................................................................................
AtDTX25     ................................................................................
AtDTX24     ................................................................................
AtDTX23     ................................................................................
AtDTX21     ................................................................................
AtDTX20     ................................................................................
AtDTX22     ................................................................................
AtDTX50     ............................................................MSQS............NRVR
AtDTX49     ..........................................................MAAPLL............MIIK
AtDTX48     ...................................................MCNSKPSSASSSL............LSCK
AtDTX53     ................................................................................
AtDTX51     ...................................................MCNPSTTTTTTGSENQESRTGLFLDLFSI
AtDTX52     ...................................................METPNIISHTN..............LLSK
AtDTX54     ............................................................................MEDK
SbBIGE1     ............................................................................MAIP
AtDTX55     ............................................................................MVVE
AtDTX56     ..............................................................................MS
```

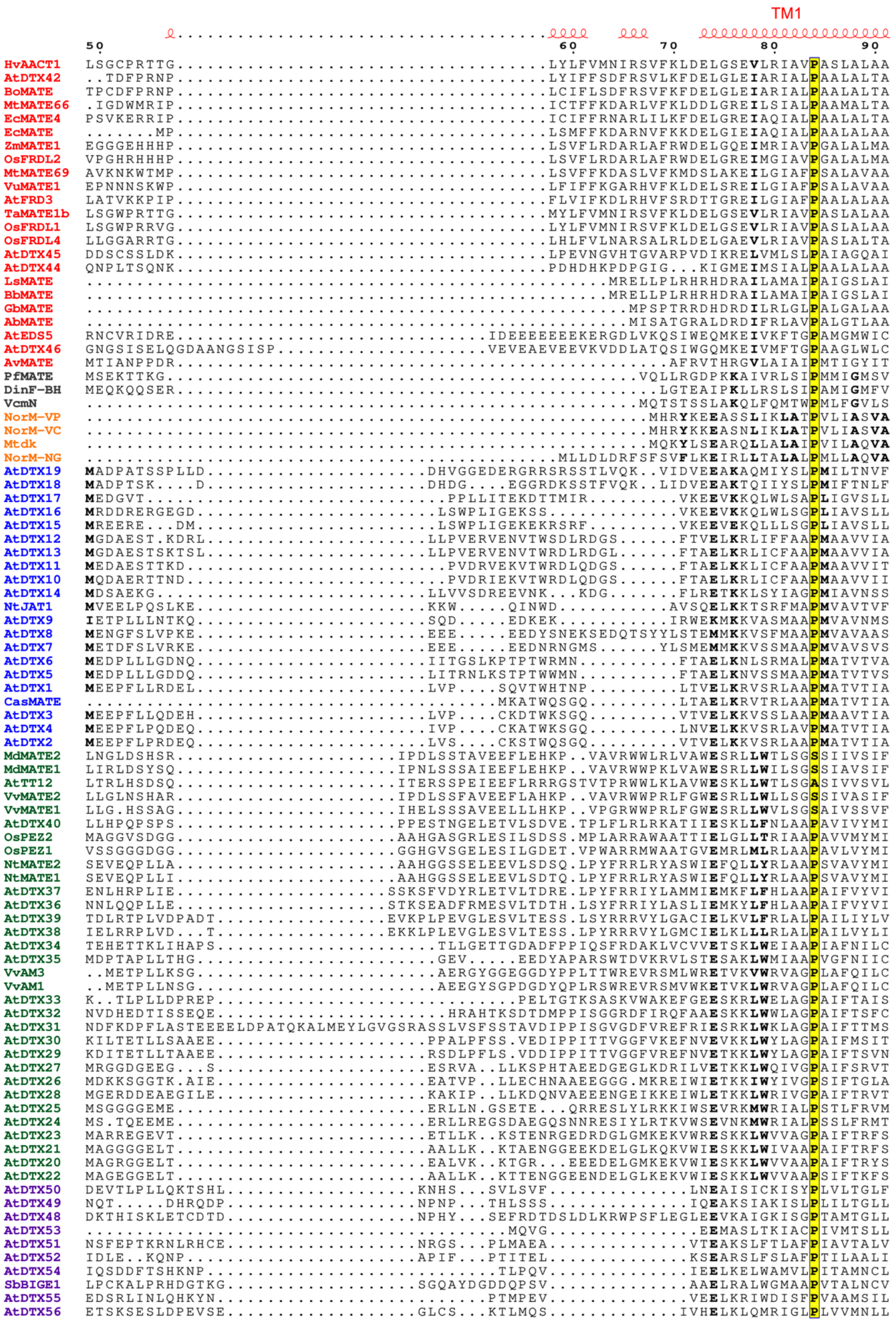
TM1
50
60
70
80
90
HvAACT1
AtDTX42
BoMATE
MtMATE66
EcMATE4
EcMATE
ZmMATE1
OsFRDL2
MtMATE69
VuMATE1
AtFRD3
TaMATE1b
OsFRDL1
OsFRDL4
AtDTX45
AtDTX44
LsMATE
BbMATE
GbMATE
AbMATE
AtEDS5
AtDTX46
AvMATE
PfMATE
DinF-BH
VcmN
NorM-VP
NorM-VC
Mtdk
NorM-NG
AtDTX19
AtDTX18
AtDTX17
AtDTX16
AtDTX15
AtDTX12
AtDTX13
AtDTX11
AtDTX10
AtDTX14
NtJAT1
AtDTX9
AtDTX8
AtDTX7
AtDTX6
AtDTX5
AtDTX1
CasMATE
AtDTX3
AtDTX4
AtDTX2
MdMATE2
MdMATE1
AtTT12
VvMATE2
VvMATE1
AtDTX40
OsPEZ2
OsPEZ1
NtMATE2
NtMATE1
AtDTX37
AtDTX36
AtDTX39
AtDTX38
AtDTX34
AtDTX35
VvAM3
VvAM1
AtDTX33
AtDTX32
AtDTX31
AtDTX30
AtDTX29
AtDTX27
AtDTX26
AtDTX28
AtDTX25
AtDTX24
AtDTX23
AtDTX21
AtDTX20
AtDTX22
AtDTX50
AtDTX49
AtDTX48
AtDTX53
AtDTX51
AtDTX52
AtDTX54
SbBIGE1
AtDTX55
AtDTX56

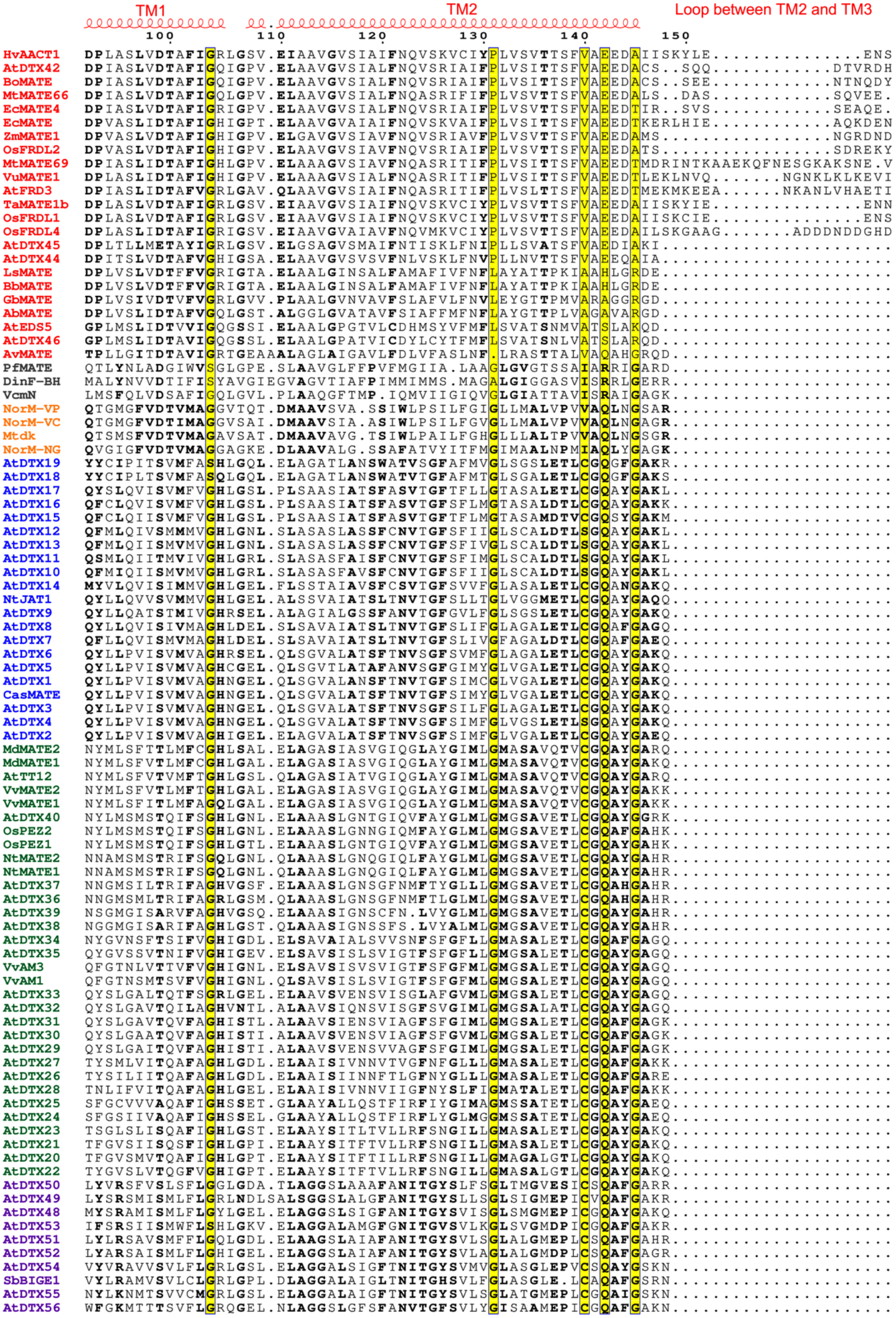
TM1
TM2
Loop between TM2 and TM3
100
110
120
130
140
150

Loop between TM2 and TM3

```
                160         170                                      180         190
HvAACT1    SQDLEKASHVHSDACNLP..........................ASGPDTPVCANSCIPTECTDLSNQG...........
AtDTX42    KECIEIGINNPTEETIEL..........................IPEKH.KDSLSDEFKTSSSIFSISKP..........
BoMATE     KECIETGINNTKEETQEL..........................IPG...NISTPDESKNSCSIFSVSDS..........
MtMATE66   NGCLEAATP.PDAETKEF..........................LPQ...KNSVVESFN......VVKDD..........
EcMATE4    SECSEACSI.ENAENKEL..........................IPRNESSDHLSESIRISSFKVSKFDQ..........
EcMATE     GDKWFPVSKEKDVEMEEL..........................LPQ...SDSTSKSSFTDTSFGKMADL..........
ZmMATE1    KIHQQNERNVSVSEMDEL..........................IPPE..GASASTSISSFETDSCEVSV..........
OsFRDL2    EINGENEFNVSDSEMEEL..........................VSHE..EASAAPSKSSFETDSSDVKI..........
MtMATE69   MPDDHLLQDIEAGATKQ......DSTLKNG...............DDANSNISKSSIVTNSG.NKSESKP..........
VuMATE1    MPEDQMLQDLEKDTLKENIKTSAKPVIGKRELKDLEVKSFGSQAVENNNATTDNNDIETGDEGNKSICKSSWITKSKEKV
AtFRD3     LVQDSLEKGISSPTSND............................TNQPQQPPAPDTKSNSG.NKSNKKE..........
TaMATE1b   SKDLEKAAHVHSDACNVP..........................ASGGDTPVCANSCIPTECADPSNQG...........
OsFRDL1    SQDLEKASPVDSETNNLP..........................VSGPDKVECVNSCIPTECTNPSDQG...........
OsFRDL4    AKGHGASAAAVADPEKQQ..........................VVGVDSAETNGAEVSTAAVRTTDDKKAAAAGVGVG.
AtDTX45    ..................................................AAQDLASEDSQSDIPSQGLP..........
AtDTX44    ..................................................AKDDNDSIETS...................
LsMATE     ................................................................................
BbMATE     ................................................................................
GbMATE     ................................................................................
AbMATE     ................................................................................
AtEDS5     ................................................................................
AtDTX46    ................................................................................
AvMATE     ................................................................................
PfMATE     ................................................................................
DinF-BH    ................................................................................
VcmN       ................................................................................
NorM-VP    ................................................................................
NorM-VC    ................................................................................
Mtdk       ................................................................................
NorM-NG    ................................................................................
AtDTX19    ................................................................................
AtDTX18    ................................................................................
AtDTX17    ................................................................................
AtDTX16    ................................................................................
AtDTX15    ................................................................................
AtDTX12    ................................................................................
AtDTX13    ................................................................................
AtDTX11    ................................................................................
AtDTX10    ................................................................................
AtDTX14    ................................................................................
NtJAT1     ................................................................................
AtDTX9     ................................................................................
AtDTX8     ................................................................................
AtDTX7     ................................................................................
AtDTX6     ................................................................................
AtDTX5     ................................................................................
AtDTX1     ................................................................................
CasMATE    ................................................................................
AtDTX3     ................................................................................
AtDTX4     ................................................................................
AtDTX2     ................................................................................
MdMATE2    ................................................................................
MdMATE1    ................................................................................
AtTT12     ................................................................................
VvMATE2    ................................................................................
VvMATE1    ................................................................................
AtDTX40    ................................................................................
OsPEZ2     ................................................................................
OsPEZ1     ................................................................................
NtMATE2    ................................................................................
NtMATE1    ................................................................................
AtDTX37    ................................................................................
AtDTX36    ................................................................................
AtDTX39    ................................................................................
AtDTX38    ................................................................................
AtDTX34    ................................................................................
AtDTX35    ................................................................................
VvAM3      ................................................................................
VvAM1      ................................................................................
AtDTX33    ................................................................................
AtDTX32    ................................................................................
AtDTX31    ................................................................................
AtDTX30    ................................................................................
AtDTX29    ................................................................................
AtDTX27    ................................................................................
AtDTX26    ................................................................................
AtDTX28    ................................................................................
AtDTX25    ................................................................................
AtDTX24    ................................................................................
AtDTX23    ................................................................................
AtDTX21    ................................................................................
AtDTX20    ................................................................................
AtDTX22    ................................................................................
AtDTX50    ................................................................................
AtDTX49    ................................................................................
AtDTX48    ................................................................................
AtDTX53    ................................................................................
AtDTX51    ................................................................................
AtDTX52    ................................................................................
AtDTX54    ................................................................................
SbBIGE1    ................................................................................
AtDTX55    ................................................................................
AtDTX56    ................................................................................
```

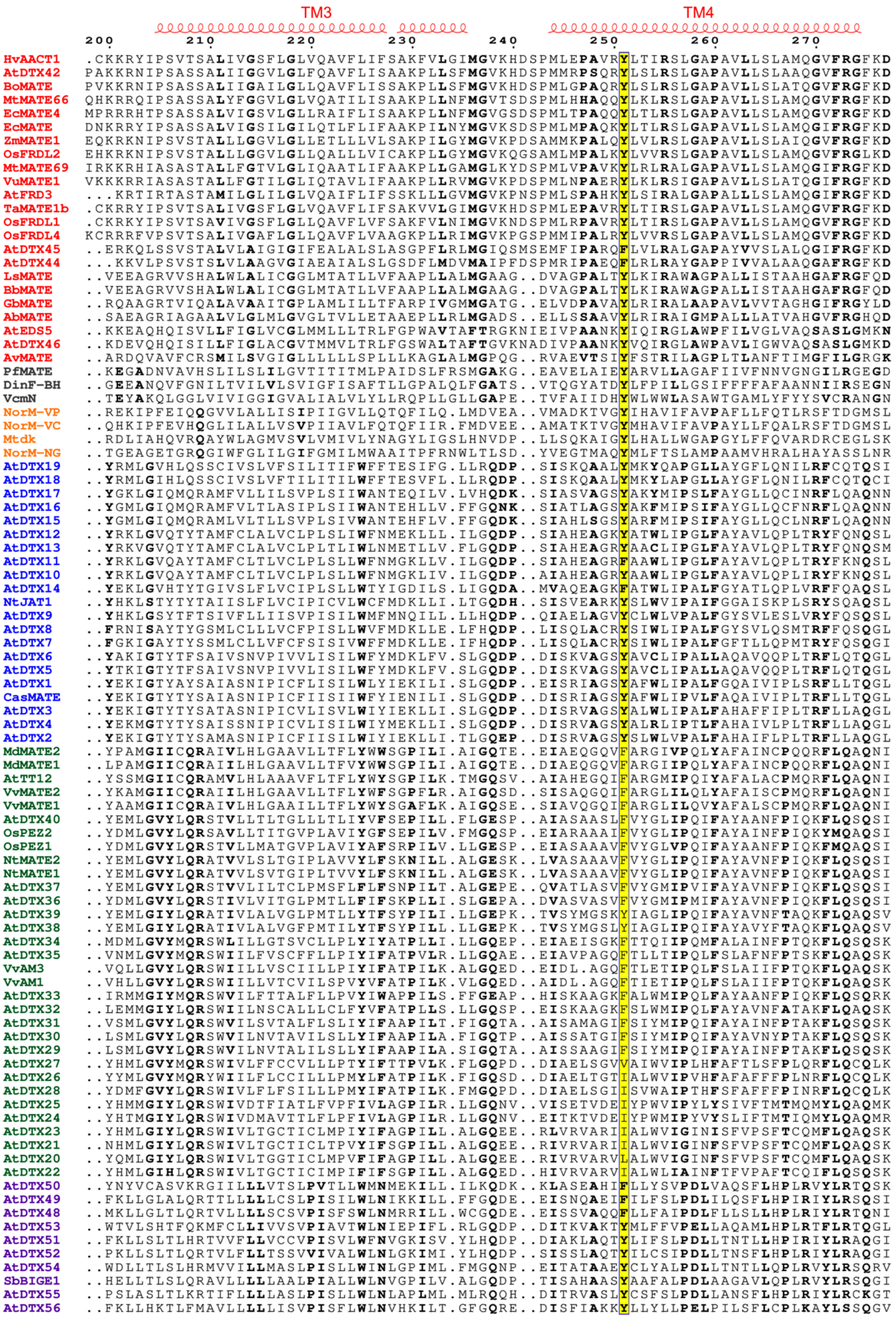
TM3
TM4
200
210
220
230
240
250
260
270
HvAACT1
AtDTX42
BoMATE
MtMATE66
EcMATE4
EcMATE
ZmMATE1
OsFRDL2
MtMATE69
VuMATE1
AtFRD3
TaMATE1b
OsFRDL1
OsFRDL4
AtDTX45
AtDTX44
LsMATE
BbMATE
GbMATE
AbMATE
AtEDS5
AtDTX46
AvMATE
PfMATE
DinF-BH
VcmN
NorM-VP
NorM-VC
Mtdk
NorM-NG
AtDTX19
AtDTX18
AtDTX17
AtDTX16
AtDTX15
AtDTX12
AtDTX13
AtDTX11
AtDTX10
AtDTX14
NtJAT1
AtDTX9
AtDTX8
AtDTX7
AtDTX6
AtDTX5
AtDTX1
CasMATE
AtDTX3
AtDTX4
AtDTX2
MdMATE2
MdMATE1
AtTT12
VvMATE2
VvMATE1
AtDTX40
OsPEZ2
OsPEZ1
NtMATE2
NtMATE1
AtDTX37
AtDTX36
AtDTX39
AtDTX38
AtDTX34
AtDTX35
VvAM3
VvAM1
AtDTX33
AtDTX32
AtDTX31
AtDTX30
AtDTX29
AtDTX27
AtDTX26
AtDTX28
AtDTX25
AtDTX24
AtDTX23
AtDTX21
AtDTX20
AtDTX22
AtDTX50
AtDTX49
AtDTX48
AtDTX53
AtDTX51
AtDTX52
AtDTX54
SbBIGE1
AtDTX55
AtDTX56

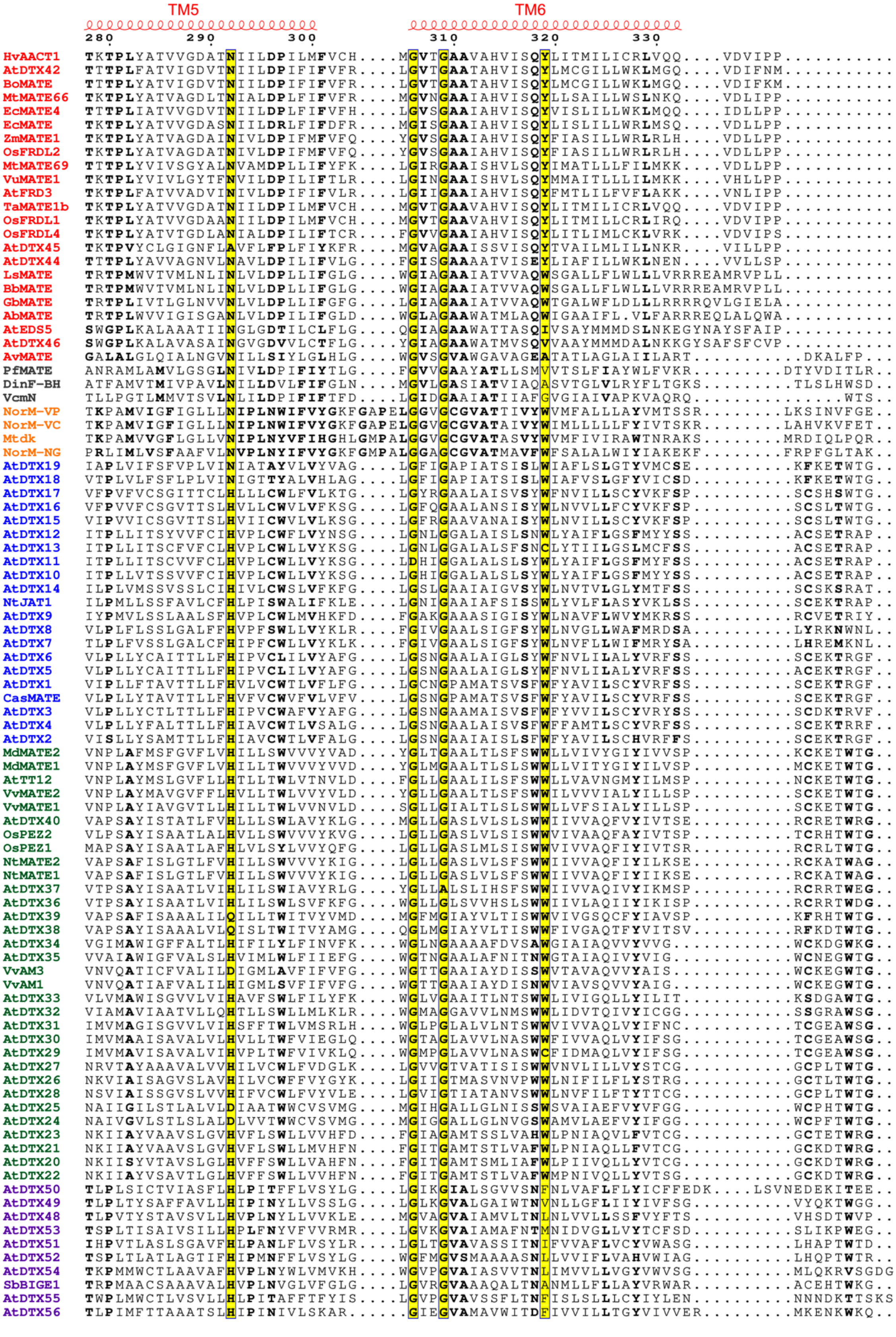
TM5
TM6
280
290
300
310
320
330

```
                                                    TM7                                    TM8
                        340         350           360           370           380           390
HvAACT1   ................SLKSLKFGRFLGCG..FLLLAR.VVAVTFCVTLASSLAARDGPTIMAAFQICCQLWLATSLLA
AtDTX42   ................STKHLQFCRFMKNG..FLLLMR.VIAVTFCVTLSASLAAREGSTSMAAFQVCLQVWLATSLLA
BoMATE    ................STKHLQFCRFMKNG..FLLLMR.VVAVTFCVTLSASLAAREGPTSMAAFQVCLQVWLATSLLA
MtMATE66  ................SIKHMQFDRFAKNGKGFLLFMR.VIAVTFCVTLSASLAAHHGSTSMAAFQVCLQVWLAVSLLA
EcMATE4   ................SFKHLQFGRFLKNG..FLLLMR.VVAVTFCVTLAASMAARLGPTSMAAFQVCLQVWLATSLLA
EcMATE    ................SIKDLKFERFLKNG..LLLLVR.VIAVTFCVTLAASLAARHGATSMAAFQVCLQIWLATSLLA
ZmMATE1   ................SFKHLQFGRFLKNG..FLLLAR.VIAATCCVTLSASMAARLGSTPMAAFQICLQTWLACSLLA
OsFRDL2   ................SFKHMQFSRFLKNG..FLLLAR.VIAATCCVTLSASMAARLGSVPMAAFQICLQIWLASSLLA
MtMATE69  ................SMKDLQIFRFLKNG..GLLLAR.VIAVTFCVTLSASLAARLGPIPMAAFQTCLQVWMTSSLLA
VuMATE1   ................SIKDLQIFRFLKNG..GLLMLR.VIAVTFCVTLAASLAARLGSIPMAAFQTCLQVWLTSSLLA
AtFRD3    ................NFGDLQFGRFLKNG..LLLLAR.TIAVTFCQTLAAAMAARLGTTPMAAFQICLQVWLTSSLLN
TaMATE1b  ................SLKSLKFGRFLGCG..FLLLAR.VVAVTSCVTLASSLAARDGPTIMAAFQICCQLWLATSLLA
OsFRDL1   ................SLKSLKFGRFLGCG..FLLLAR.VVAVTFCVTLASSLAARHGPTIMAAFQICCQLWLATSLLA
OsFRDL4   ................SLKSLKFRRFLGCG..FLLLAR.VVAVTFCVTLAASLAARHGATAMAAFQICAQVWLASSLLA
AtDTX45   ................KIGSLKFGDYLKSG..GFVLGR.TLSVLVTMTVATSMAARQGVFAMAAHQICMQVWLAVSLLT
AtDTX44   ................QIKVGRANQYLKSG..GLLIGR.TVALLVPFTLATSLAAQNGPTQMAGHQIVLEIWLAVSLLT
LsMATE    ................APSLSGMLPLVRVG..SALLLR.TGALVGTMTLATAVAARQGAESVAAHQVANQLWGFFALLI
BbMATE    ................APSLSGMLPLVRVG..SALLLR.TGALVGTMTLATAVAARQGAESVAAHQVANQLWGFFALLI
GbMATE    ................LPRLVELMPFLRIG..GALALR.TFALIGTMTVATAVATRVGTVAVAAHLVATEIWLFLALVV
AbMATE    ................WPKREALGPLVGAG..RALVIR.SGALLTAFTLATAVATRQGEEVVAAHQVAVQLWIFLALVV
AtEDS5    ................SPQELWKISALAAP....VFIS.IFSKIAFYSFIIYCATSMGTHVLAAHQVMAQTYRMCNVWG
AtDTX46   ................SPSELLTIFGLAAP....VFIT.MMSKVLFYTLLVYFATSMGTNIIAAHQVMLQIYTMSTVWG
AvMATE    ...............SLAELLDRNKLAQLFALNRDILIRSFVLLAAFTVLTRIGTGFGAVTLAANALLMNFFMIASFYL
PfMATE    ...............DFSPSREILKDILRVGLPSSLSQL..SMSIAMFFLNSVAITAGGENGVAVFTSAWRITMLGIVPI
DinF-BH   ..............LRMKGSVIKEVCLVGLPAFVQQS..SASLMMIAINSMLLRFGSDFYVGVFGLVQRIMMFVMMPM
VcmN      ..............YQWQDLNISQSLTALGHIMGPAMLSQLLPPLSSMFATKLLASFGTAAVAAWALGSRFEFFALVAV
NorM-VP   ..............YHKPQWKAQVRLFKLGFPVAAALF.FEVTLFAVVALLVSPLG..PIIVAAHQVAINFSSLVFMLP
NorM-VC   ..............FHKPQPKELIRLFRLGFPVAAALF.FEVTLFAVVALLVAPLG..STVVAAHQVALNFSSLVFMFP
Mtdk      ..............FSAPDWKAIGRLTGLGLPVALALF.FEVTLFAVVALLVSPLG..IVNVAGHQIALNFSSLMFVVP
NorM-NG   ..............FGKPDWAVFKQIWKIGAPIGLSYF.LEASAFSFIVFLIAPFG..EDYVAAQQVGISLSGILYMIP
AtDTX19   ..............FSLESFRYIVINLTLSLPSAAMVC.LEYWAFEILVFLAGVMPNPEINTSLVAICVNTEAISYMLT
AtDTX18   ..............FSMESFHHVVLNLTLSIPSAAMVC.LEYWAFEILVFLAGLMRNPEITTSLVAICVNTESISYMLT
AtDTX17   ..............FSKEAFQELYDFSKIAFPSAVMVC.LELWSFELLVLASGLLPNPVLETSVLSICLNTSLTIWQIS
AtDTX16   ..............FSKEALRDILPFLRLAVPSALMVC.LEMWSFELLVLLSGLLPNPVLETSVLSICLNTSGTMWMIP
AtDTX15   ..............FSKEARRDIIPFMKLVIPSAFMVCSLEMWSFELLVLSSGLLPNPVLETSCP.......RTVWMIP
AtDTX12   ..............LSMEIFDGIGEFFKYALPSAAMIC.LEWWSYELIIILSGLLPNPQLETSVLSVCLQTISTMYSIP
AtDTX13   ..............LSMEIFDGIGEFFRYALPSAAMIC.LEWWSYELIIILSGLLPNPQLETSVLSVCLQTTATVYSIH
AtDTX11   ..............LTMEIFEGVREFIKYALPSAAMLC.LEWWSYELIIILSGLLPNPQLETSVLSVCLQTLSMTYSIP
AtDTX10   ..............LTMEIFEGVREFIKYALPSAAMLC.LEWWSYELIIILSGLLPNPQLETSVLSICFETLSITYSIP
AtDTX14   ..............ISMSLFEGMGEFFRFGIPSASMIC.LEWWSFEFLVLLSGILPNPKLEASVLSVCLSTQSSLYQIP
NtJAT1    ..............FSMEAFLCIRQFFRLAVPSAVMVC.LKWWSFEVLALVSGLLPNPKLETSVMSICITISQLHFSIP
AtDTX9    ..............MSKDVFVHTNIFFQFAIPSAMMCC.LEWLAFEVITLLSGLLPNSKLETSVISICLTTSSLHYNLV
AtDTX8    ..............RAQEIFLSMKQFITLAIPTAMMTC.LEWWSFELLILMSGLLPNSKLETSVLSICLTMSSLHYVIV
AtDTX7    ..............GLQELISSMKQFIALAIPSAMMIC.LEWWSFEILLMSGLLPNSKLETSVISICLTTSAVHFVLV
AtDTX6    ..............VSDDFVLSVKQFFQYGIPSAAMTT.IEWSLFEFLILSSGLLPNPKLETSVLSICLTTSSLHYVIP
AtDTX5    ..............VSDDFVLSVKQFFQYGIPSAAMTT.IEWSLFELLILSSGLLPNPKLETSVLSICLTTSSLHCVIP
AtDTX1    ..............VSRDFVSSIKQFFQYGIPSAAMIC.LEWWLFEILILCSGLLPNPKLETSVLSICLTIETLHYVIS
CasMATE   ..............VSEDFVSCVKQFFQYGVPSAAMIC.LEWWLFELLILCSGLLSNPKLETSVLSICLTTETLHYVIS
AtDTX3    ..............VSSDFVSCIKQFFHFGVPSAAMVC.LEWWLFELLILCSGLLPNPKLETSVLSICLTTASLHYVIP
AtDTX4    ..............VSQDFLSSVKQFFRYGVPSAAMLC.LEWWLFELLILCSGLLQNPKLETSVLSICLTTATLHYVIP
AtDTX2    ..............VSNDFMSSIKQYFQYGVPSAGLIC.LEWWLFELLILCSGLLPNPKLETSVLSICLTIGTLHYVIP
MdMATE2   ..............FSGKALWGIWPYFKLTVASAIMLC.LEIWYSQGLVLISGLLPNPTIALDSISICMNYLNWDMQFM
MdMATE1   ..............FSWKAFRGIWPYFKLTLASAIMLC.LEIWYNQGLVLISGLLSNPTISLDSISICMNYLNWDMQFM
AtTT12    ..............FSTRAFRGIWPYFKLTVASAVMLC.LEIWYNQGLVIISGLLSNPTISLDAISICMYYLNWDMQFM
VvMATE2   ..............FSSKAFKGMWPYFKLTVASAVMLC.LEIWYNQGLVLISGLLSNPTISLDSISICMNYLNWDMQFM
VvMATE1   ..............FSLKAFQGIWPYFKLTVASAVMLC.LEIWYSQGLVLISGLLPNPTVSLDSISICMNYLNWDITFM
AtDTX40   ..............FSVQAFSGLWSFFKLSAASAVMLC.LETWYFQILVLLAGLLENPELALDSLSICMTISGWVFMIS
OsPEZ2    ..............FTWQAFAGLWDFLKLSAASAVMLC.LESWYFQVLVLIAGLLPNPELALDALSVCMTISGWVFMIS
OsPEZ1    ..............FSMLAFSGLPDFFKLSLASAVMLC.LETWYFQILVLIAGLLKDPEMALASLSVCMTISGWVFMIS
NtMATE2   ..............FRWEAFSGLWQFVKLSAGSAVMLC.LETWYFQILVLLSGLLKNPEIALASISVCLAVNGLMFMVA
NtMATE1   ..............FRWEAFSGLCQFVKLSAGSAVMLC.LETWYMQILVLLSGLLKNPEIALASISVCLAVNGLMFMVA
AtDTX37   ..............FSWKAFEGLWDFFRLSAASAVMLC.LESWYSQILVLLAGLLKNPELALDSLAICMSISAISFMVS
AtDTX36   ..............FSWKAFDGLWDFFQLSAASAVMLC.LESWYSQILVLLAGLLKDPELALDSLAICMSISAMSFMVS
AtDTX39   ..............LSWRSLQGLWSFFKLSAGSAVMLC.LEMWYSQILVLLAGLLENPARSLDSLSICMSISALSFMVS
AtDTX38   ..............FSWKSLHGLWSFFKLSAGSAVMIC.LEIWYTQILVLLAGLLKDPALSLDSLSICMSISALSFMVS
AtDTX34   ..............LSWLAFQDVWPFLKLSFASAVMLC.LEIWYFMTIIVLTGHLEDPVIAVGSLSICMNINGWEGMLF
AtDTX35   ..............LSWLAFKEIWAFVRLSIASAVMLC.LEIWYMMSIIVLTGRLDNAVIAVDSLSICMNINGLEAMLF
VvAM3     ..............LTWSAFREIWAFVRLSLASAVMLC.LEIWYFMIIILLTGHLQNAVIAVGSLSICMTFGGLEVVMF
VvAM1     ..............LTWSAFREIWAFVRLSIASAVMLC.LEIWYFTIIVLLTGHLQNAVIAVGSLSICMTFNGWEGMLF
AtDTX33   ..............FSMLAFRDLYGFVKLSLASALMLC.LEFWYLMVLVVVTGLLPNPLIPVDAISICMNIEGWTAMIS
AtDTX32   ..............LSWMAFKNLRGFARLSLASAVMVC.LEVWYFMALILFAGYLKNPQVSVAALSICMNILGWPIMVA
AtDTX31   ..............FTWEAFHNLWGFVKLSLASAAMLC.LEIWYFMALVLFAGYLKNAEVSVAALSICMNILGWAAMVA
AtDTX30   ..............FSWEAFHNLWSFVRLSLASAVMLC.LEVWYLMAVILFAGYLKNAEISVAALSICMNILGWTAMIA
AtDTX29   ..............FSWEAFHNLWSFVRLSLASAVMLC.LEVWYFMAIILFAGYLKNAEISVAALSICMNILGWTAMIA
AtDTX27   ..............LSSEALTGLWEFLKLSASSGVMLC.LENWYYRILIIMTGNLQNARIAVDSLSICMAINGWEMMIP
AtDTX26   ..............FSSEAFTGLLELTKLSASSGIMLC.LENWYYKILMLMTGNLVNAKIAVDSLSICMSVNGWEMMIP
AtDTX28   ..............FSMESFTRLWEFTKLSASSGIMVC.LENWYYRMLIVMTGNLEDARIDVDSMSICMSINGLEMMVP
AtDTX25   ..............FSTAAFLDLIPMLKLSISSGFMLC.LEYWYMSIIVLMSGYAKDANIAISAFSICQYIYSWEMNIC
AtDTX24   ..............FSIAAFVDLIPMLKLSISSGFMIC.LEYWYMSILVLMAGYTKDAKIAISAFSICQYIYTWELNIC
AtDTX23   ..............FSWLAFKDLWPVFKLSVSSGGMIC.LELWYNSILILLTGNLKNAEVALNALAICININALEMMVA
AtDTX21   ..............FSMMAFKDLWPVFKLSMSSGGMLC.LELWYNSILVLLTGNLKNAEVALDALAICLNINGLEMMIA
AtDTX20   ..............FSMLAFKDLWPVLKLSLSSGGMLC.LELWYNSVLVLLTGNLKNAEVALDALAICISINALEMMIA
AtDTX22   ..............FTMLAFKDLWPVFKLSLSSGGMVC.LELWYNSILVLLTGNLKNAEVAIDALAICINVNALQMMIA
AtDTX50   ............TCE.DSVREWKKLLCLAIPSCISVC.LEWWCYEIMILLCGFLLDPKASVASMGILIQITSLVYIFP
AtDTX49   ............FSM.DCFKGWRSLMKLAIPSCVSVC.LEWWWYEIMILLCGLLLNPQATVASMGILIQTTALIYIFP
AtDTX48   ............ITI.DSLKGWSALLSLAIPTCVSVC.LEWWWYEFMIILCGLLANPRATVASMGILIQTTALVYVFP
AtDTX53   ............LALRSLFRGWWPLLSLAAPSAISVC.LEYWWYEIMLFLCGLLGNPKASVAAMGILIQTTGILYVVP
AtDTX51   ............PTR.DCFRGWAPLLRLAGPSCVSVC.LEWWWYEIMIVLCGLLVNPRSTVAAMGVLIQTTSFLYVFP
AtDTX52   ............PSS.ECFKDWGPVVTLAIPSCIGVC.LEWWWYEIMTVLCGLLIDPSTPVASMGILIQTTSLLYIFP
AtDTX54   DGGSTTMVAVVAQSSSVMELVGGLGPLMRVAVPSCLGIC.LEWWWYEIVIVMGGYLENPKLAVAATGILIQTTSLMYTVP
SbBIGE1   ..........WARPADVAAGLPALAGLAVPSCVGVC.LEWWWYEVVTVLAGYLPNPAAAVGAAGVLIQTTSLMYTVP
AtDTX55   LCLDTP...LMLYGSRDSGENDVWSTLVKFAVPSCIAVC.LEWWWYEFMTVLAGYLPEPKVALAAAAIVIQTTSLMYTIP
AtDTX56   ............GGWLNQSAQDWLTLIKLSGPCCLTVC.LEWWCYEILVLLTGRLPNPVQAVSILIIVFNFDYLLYAVM
```

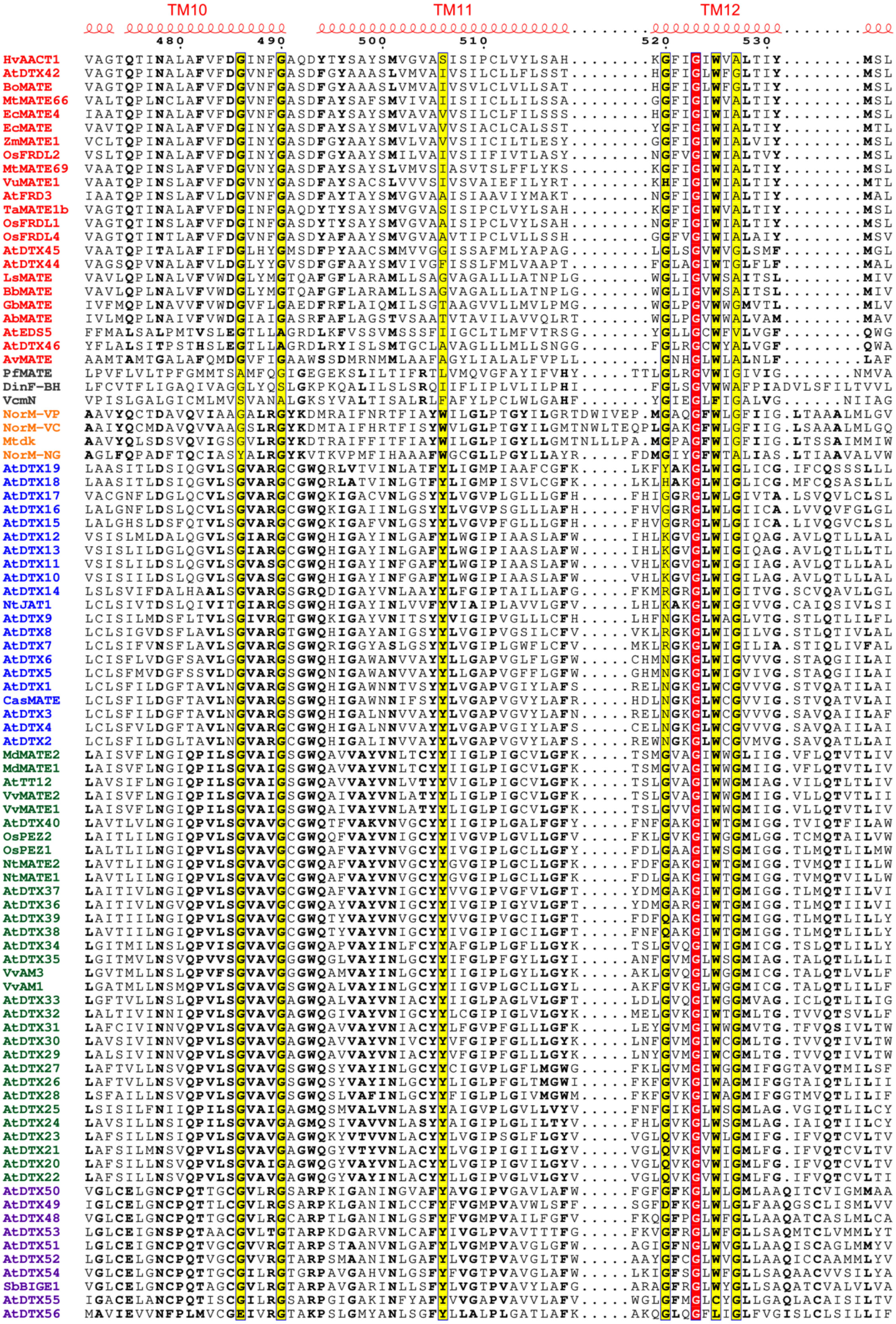
TM10
TM11
TM12
480
490
500
510
520
530
HvAACT1
AtDTX42
BoMATE
MtMATE66
EcMATE4
EcMATE
ZmMATE1
OsFRDL2
MtMATE69
VuMATE1
AtFRD3
TaMATE1b
OsFRDL1
OsFRDL4
AtDTX45
AtDTX44
LsMATE
BbMATE
GbMATE
AbMATE
AtEDS5
AtDTX46
AvMATE
PfMATE
DinF-BH
VcmN
NorM-VP
NorM-VC
Mtdk
NorM-NG
AtDTX19
AtDTX18
AtDTX17
AtDTX16
AtDTX15
AtDTX12
AtDTX13
AtDTX11
AtDTX10
AtDTX14
NtJAT1
AtDTX9
AtDTX8
AtDTX7
AtDTX6
AtDTX5
AtDTX1
CasMATE
AtDTX3
AtDTX4
AtDTX2
MdMATE2
MdMATE1
AtTT12
VvMATE2
VvMATE1
AtDTX40
OsPEZ2
OsPEZ1
NtMATE2
NtMATE1
AtDTX37
AtDTX36
AtDTX39
AtDTX38
AtDTX34
AtDTX35
VvAM3
VvAM1
AtDTX33
AtDTX32
AtDTX31
AtDTX30
AtDTX29
AtDTX27
AtDTX26
AtDTX28
AtDTX25
AtDTX24
AtDTX23
AtDTX21
AtDTX20
AtDTX22
AtDTX50
AtDTX49
AtDTX48
AtDTX53
AtDTX51
AtDTX52
AtDTX54
SbBIGE1
AtDTX55
AtDTX56

```
                TM12
               540       550
HvAACT1   RTVASTWRMGAARGPWVFLRK.....................................
AtDTX42   RAAVGFWRIGTGTGPWSFLRS.....................................
BoMATE    RAAVGFWRIGTATGPWSFLRR.....................................
MtMATE66  RAFAGFLRIGTGSGPWEFLRS.....................................
EcMATE4   RAFAGFWRIGTGTGPWNFLWG.....................................
EcMATE    RTFAGFLRIGAGMGPWGFLNN.....................................
ZmMATE1   RMFAGFWRIGTAQGPWAYLRG.....................................
OsFRDL2   RMLAGFLRIGTARGPWTFYAAQRMHSHEVVGLC.........................
MtMATE69  RMFAGVWRMGTGTGPWRFLRGQSLS.................................
VuMATE1   RMMAGVWRMGTGTGPWRYLRGCSLA.................................
AtFRD3    RAITGIARMATGTGPWRFLRGRSSSSSS..............................
TaMATE1b  RTVASTWRMGAARGPWAFLRK.....................................
OsFRDL1   RTIASTWRMGAARGPWVFLRK.....................................
OsFRDL4   RAFASTWRMGAARGPWKFLRK.....................................
AtDTX45   RMVAGFSRLMWRKGPWWFMHTSDKRLA...............................
AtDTX44   RLVAGAWRLGTRTGPWKMLWSAPEKPE...............................
LsMATE    RGLTLGVPWATRRVPGLD........................................
BbMATE    RGLTLGVPWATRRVPGLD........................................
GbMATE    RGVTLG..WRYWRAWPTN........................................
AbMATE    RFGTLAWWHVTGPLSSGAGPSPGFPAAG..............................
AtEDS5    RFGLYLRRLLSPGGILNSDGPSPYTVEKIKSI..........................
AtDTX46   RFSLSLFRLLSRDGVLYSEDTSRY.AEKVKAA..........................
AvMATE    RGLFLALRLKSRRDQTFSARQ.....................................
PfMATE    AIVGFLWGRMRISALKKTSATGGKR.................................
DinF-BH   LYRDRNVFFLKTKEERELDLVKTASST...............................
VcmN      WAAWLAYQKALRQLEGAHHTSA....................................
NorM-VP   RLRWMHRQEPDVQLNFSLQ.......................................
NorM-VC   RLYWLQKQSDDVQLHLAAK.......................................
Mtdk      RMRRLQRQPNATVLARAAR.......................................
NorM-NG   CLEKYSMELVKSHKAV..........................................
AtDTX19   MTIFRKWTKLNVATV...........................................
AtDTX18   MTIFRKWTKLTAATV...........................................
AtDTX17   VTIFTNWDKEAKKATNRVGSS.DDKDGDVQ............................
AtDTX16   VTIFTNWDEEAKKATNRIESSSSVKDFAVDDRSVVVF.....................
AtDTX15   ITFFTNWDEEVKKATSRAKSSSEVKEFAVDNGSILV......................
AtDTX12   VTGCTNWESQADKARNR.MALAYGT.................................
AtDTX13   VTGCTNWESQADKARNR.MALAYGT.................................
AtDTX11   VTGCINWENQAREARKR.MAVAHESELTESELPF........................
AtDTX10   VTGCTNWKTQAREARER.MAVAHESELTESELPI........................
AtDTX14   IVILTNWKKQARKARERVMGDEYEEKESEEEHEYIS......................
NtJAT1    VTGFTDWEKQAKKARERVHEGRS...................................
AtDTX9    VIGFTNWSKEAIKARERIGDEKVWR....HDSLLN.......................
AtDTX8    VTFFTNWEQEVAKARDRVIEMIPQ......EII.........................
AtDTX7    VTFFTNWEQEATKARDRVFEMTPQVKGNQKTQIIVEEDTQVLLNHIAETV........
AtDTX6    VTACMSWNEQAAKARQRIVVRTSSFGNGLA............................
AtDTX5    VTACLSWEEQAAKARERIVGRTLE..................................
AtDTX1    VTASINWKEQAEKARKRIVSTENRLA................................
CasMATE   VTASMNWKEQAEKARKRIV.......................................
AtDTX3    VTASINWKEQAEKARKRMVSSENRLA................................
AtDTX4    VTASMNWKEQAKKARKRLISSENGLA................................
AtDTX2    VTASMNWKEQAEKARKRIISTENGLV................................
MdMATE2   LTARTNWTAEVEKAAERLKRSASAERLKRSASAERLDLVTDI................
MdMATE1   LTARTNWDSEVVKAAERLK.........KSASAERLDLVTDI................
AtTT12    LTLKTNWTSEVENAAQRVK..........TSATENQEMANAGV...............
VvMATE2   LTARTDWNAEVSKAAERLR.........NSANVENLNLLEDV................
VvMATE1   LTARTNWDAEVVKAVDRIK.........KSSNEETLDLVLDKI...............
AtDTX40   VTFRTDWTKEVEEASKRLD.........KWSNKK.QEVVPE.................
OsPEZ2    VTLRTDWNNEVEEAQKRLN.........KWEDKKKEPLLTGIRDNN............
OsPEZ1    VTFRTNWNREVEEAMKRLN.........KWEDKT..PLLSE.................
NtMATE2   VTFSTDWNKEVESARKRLD.........KWENLK.GPLNKEEFPGENLYFQ.......
NtMATE1   VTFRTDWNKKVECAKKRLD.........KWENLK.GPLNKE.................
AtDTX37   VTLRTDWDKEVEKASSRLD.........QWEESR.EPLLKQ.................
AtDTX36   VTFRTDWDKEVEKASRRLD.........QWEDTS..PLLKQ.................
AtDTX39   VTYRTDWDKEVEKARKRLD.........LWDDKK.EPLQN..................
AtDTX38   VTYQADWDKEVEKARKRLD.........MWDDK..EPLQN..................
AtDTX34   MIYITNWNKEVEQASERMK.........QWGAGYEK....LEKIAT............
AtDTX35   VLYKTNWNKEVEETMERMK.........KWGGSETT....SKDILA............
VvAM3     IVYRTNWNREVEQTTERMQ.........KWGGQRIE....ADDV..............
VvAM1     IVYRTNWNKEVEQTTERMQ.........KWGGVQIETKKTSDDV..............
AtDTX33   IIYFTNWNKEAEQAESRVQ.........RWGGTAQE......................
AtDTX32   IIYRTNWKKEASLAEARIK.........KWGDQSNKREEIDLCEEDENNSNGENNHRK
AtDTX31   MICKTNWEKEASMAEERIK.........EWGGVPAEKETLLN................
AtDTX30   MICRTNWDTEAAMAEGRIR.........EWGGEVSD..QLLN................
AtDTX29   MICKTNWDTEASMAEDRIR.........EWGGEVSEIKQLIN................
AtDTX27   ITMRCDWEKEAQKASARIN.........KWSN.TIK......................
AtDTX26   ITTRCDWDNEAHKSSVRIK.........KWLV.SDAGN....................
AtDTX28   ITMRCDWEKEAQNAKVRVN.........KWSV.SDARK....................
AtDTX25   VIYKTDWELEVKKTNERMK.........TWTLNLPAVQSTTISTRDEERK........
AtDTX24   IIYKTDWELEVKRTCERMK.........VWSLKPSNEESNPIIREESRSK........
AtDTX23   MTMRTDWDQQVSSSLKRLN.........RWVE.PESPSRNQTLQNE............
AtDTX21   MTLRTDWDQQVSTSLRRLN.........RWVV.PESRDVNQVSSEE............
AtDTX20   MTLRTDWDQQVSTSLRNIN.........RWVV.PESRDANQISSEE............
AtDTX22   MTLRTDWDQQVSTSLKNIN.........RWVV.PESRDANQISSEE............
AtDTX50   TCR.TDWELEAERAKVLTTAVDCGSSD....DDAKEDMEAGMVDK.............
AtDTX49   LAR.TDWEVEVHRAKELMTRSCDGDE.....DDGNTPFLLDSLDIEENLVF.......
AtDTX48   LLR.TDWKVQAERAEELTSQTPGKSPPLLPIASSKSRSTSGTEDMMRTMLV.......
AtDTX53   LIR.TDWSHQVKRAEELTSAAADKSHSEDETVHAEVQDDDDVSSNDLEIGLLQNTN..
AtDTX51   VGT.TDWESEAKKAQTLTCAETVENDIIKAVVASTIDGECDEAEPLIRITVLY.....
AtDTX52   VAT.TDWEKEAIRARKLTCTEGVD......VVITTTQTNGDLSEPLIYVVTVATD...
AtDTX54   VLARTDWEGEAVKAMRLTSLEMRKVG.....QDEESSLLLLDDEKLGDVL........
SbBIGE1   VVWRTDWRVEAMRAKKLAGLELTNDA.....EEESRRLVAATGEQAEDTS........
AtDTX55   VYN.TDWNKESLKAHDLVGKNVISPN.....VDQ...IIVKCEEGLH...........
AtDTX56   IAR.IDWEKEAGKAQILTCNTEDEQTS...........QGSGQDSHS...........
```

Fig. S13. Full sequence alignment of 94 MATE transporters.

The multiple sequence alignment was perfomed by ClustalW and viewed by ESPript 3.0. Each protein name is color-coded as follows: CAT-eMATE (eMATE3) in red, DinF subfamily in black, NorM subfamily in organge, eMATE1 in blue, eMATE2 in green, eMATE4 in purple. Residues that are strictly conserved across all groups were shown in white on a red backround. Residues that are similar or conserved within a group are displayed in bold black letters. Residues that are conserved within a group but across different groups are highlited with a yellow box. The secondary structure of $HvAACT1_{cryst}$ is labeled at the top, along with its corresponding amino acid numbers.

Fig. S14. Molecular Dynamics simulations of the inward-facing AlphaFold2-predicted model of HvAACT1$_{cryst}$.

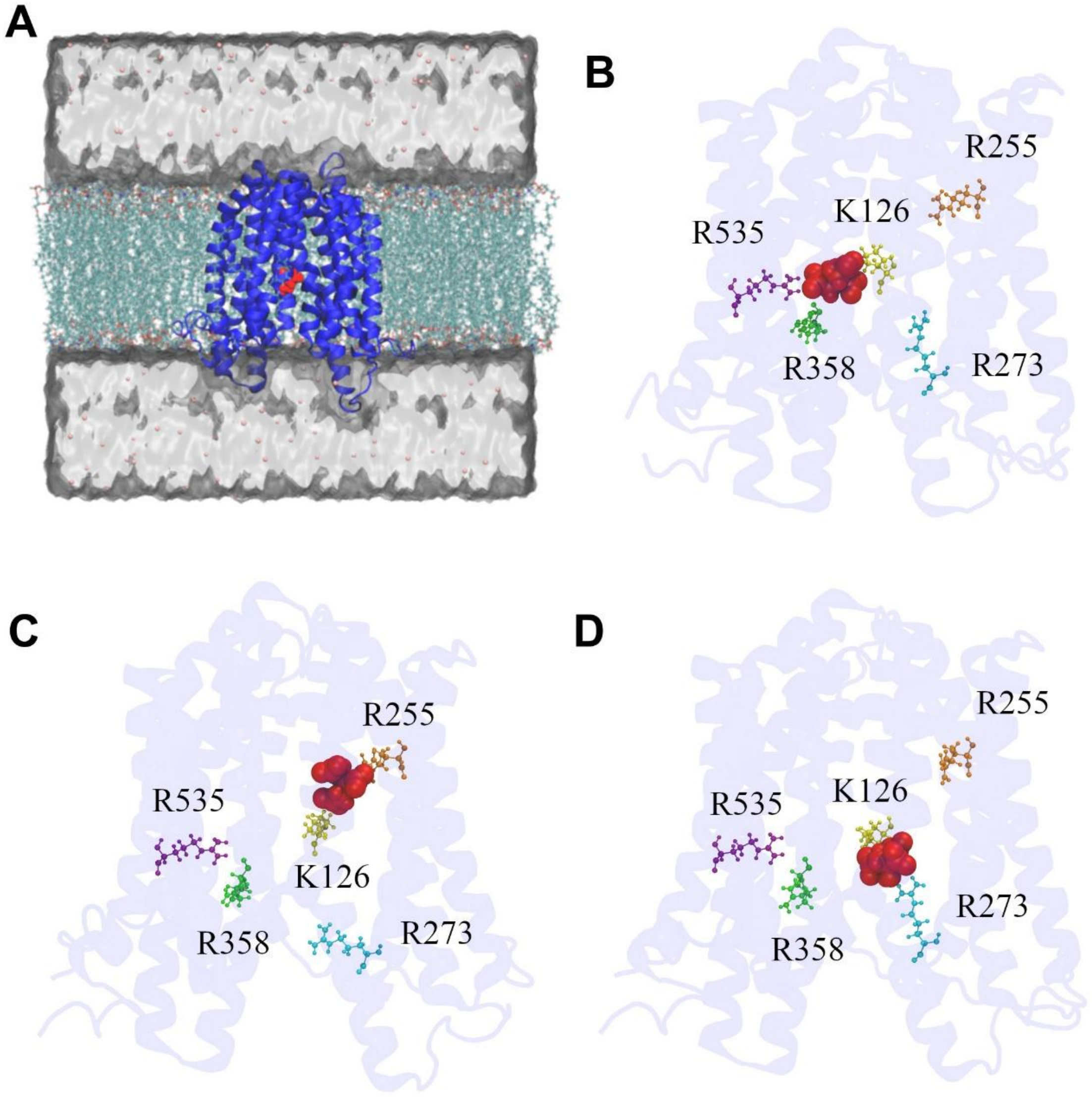

(A) Snapshot of all-atom MD system. HvAACT1$_{cryst}$ (blue) embedded in a POPC membrane (cyan) and water (red). **(B-D)** Three representative stable citrate binding modes identified by the MD simulations. The protein is shown in blue, with citrate in red and key interacting residues highlighted: K126 (yellow), R255 (orange), R273 (cyan), R358 (green), and R535 (purple). Binding modes where citrate forms stable interactions **(B)** with K126, R358, and R535, **(C)** with K126 and R255, and **(D)** with K126 and R273.

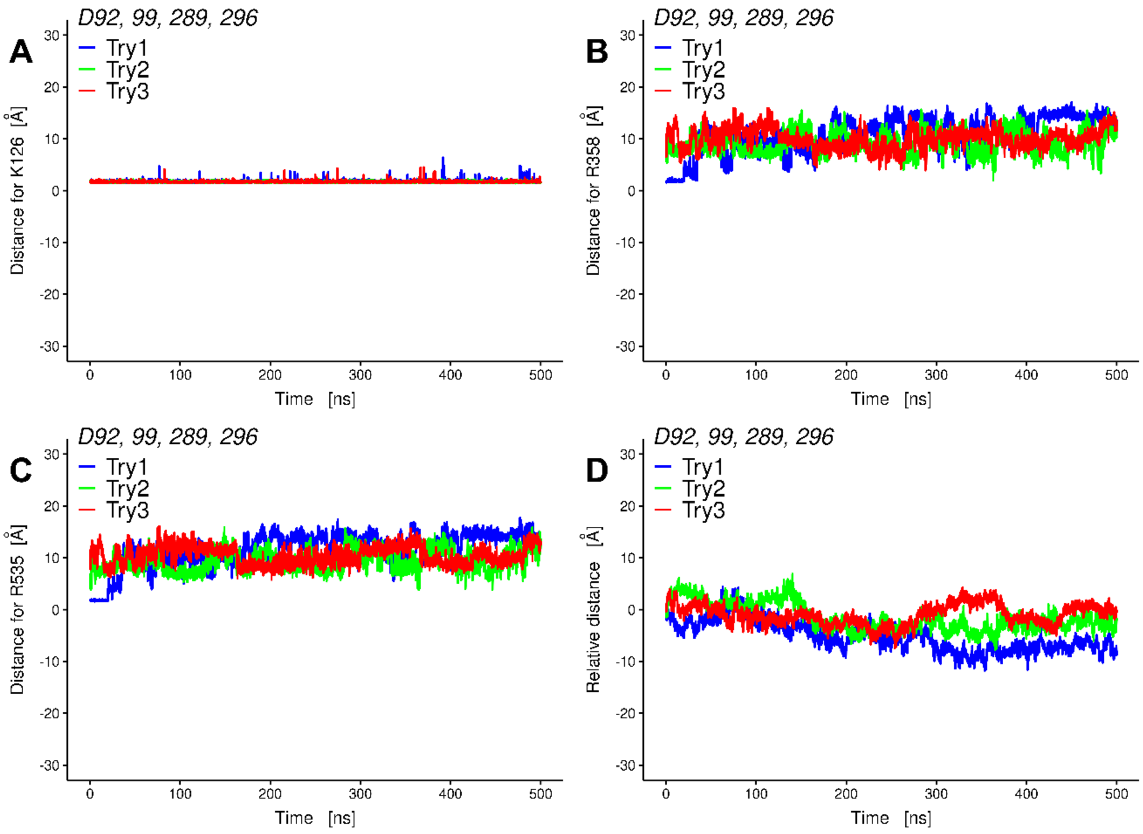

Fig. S15. Time series data of the condition in which four Asp residues are protonated (D92, D99, D289, and D296) from three independent simulations (Try1, Try2, Try3).

(A-C) The center-of-mass distances between citrate and **(A)** K126, **(B)** R358, and **(C)** R535, over 500 ns, throughout all simulations. **(D)** The z-coordinate position of citrate's center of mass relative to the membrane center was calculated from the average position of POPC phosphorus atoms. **(A-D)** All distances are measured in Å as a function of simulation time.

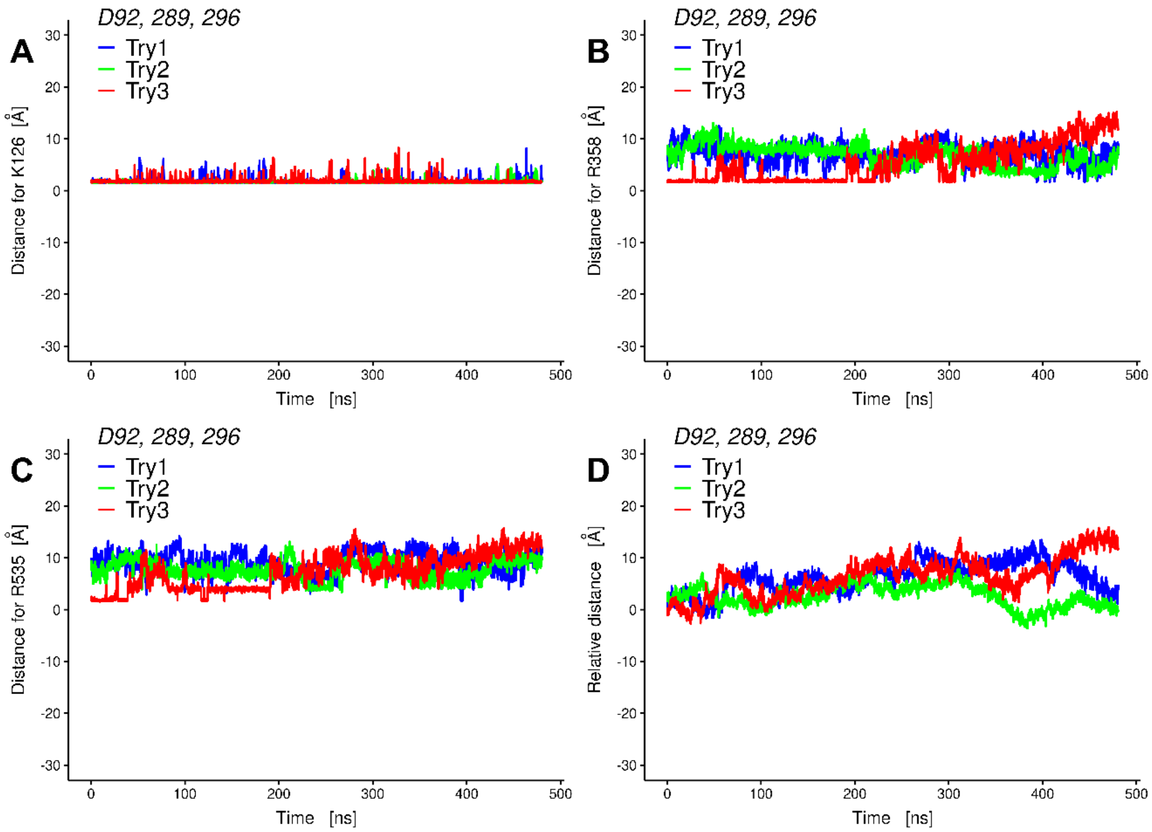

Fig. S16. Time series data of the condition in which three Asp residues are protonated (D92, D289, and D296) from three independent simulations (Try1, Try2, Try3).

(A-C) The center-of-mass distances between citrate and **(A)** K126, **(B)** R358, and **(C)** R535, over 500 ns, throughout all simulations. **(D)** The z-coordinate position of citrate's center of mass relative to the membrane center was calculated from the average position of POPC phosphorus atoms. **(A-D)** All distances are measured in Å as a function of simulation time.

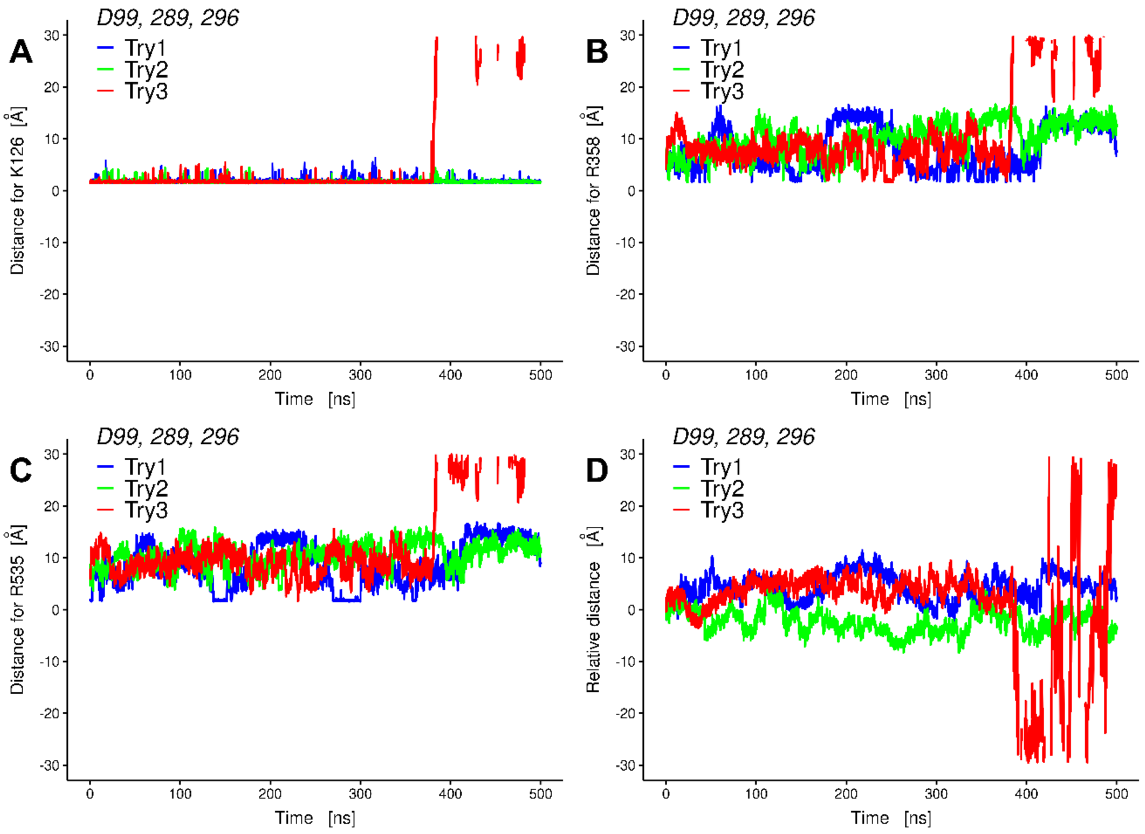

Fig. S17. Time series data of the condition in which three Asp residues are protonated (D99, D289, and D296) from three independent simulations (Try1, Try2, Try3).

(A-C) The center-of-mass distances between citrate and **(A)** K126, **(B)** R358, and **(C)** R535, over 500 ns, throughout all simulations. **(D)** The z-coordinate position of citrate's center of mass relative to the membrane center was calculated from the average position of POPC phosphorus atoms. **(A-D)** All distances are measured in Å as a function of simulation time.

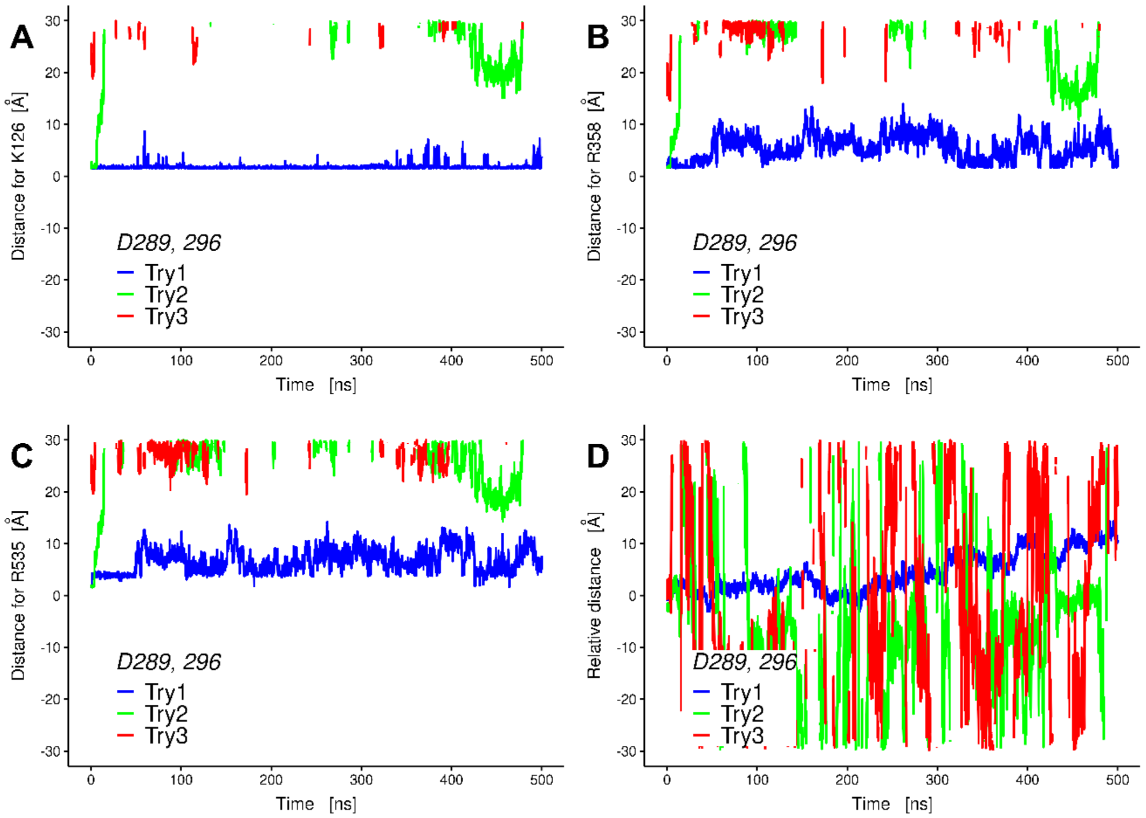

Fig. S18. Time series data of the condition in which two Asp residues are protonated (D289 and D296) from three independent simulations (Try1, Try2, Try3).

(A-C) The center-of-mass distances between citrate and **(A)** K126, **(B)** R358, and **(C)** R535, over 500 ns, throughout all simulations. **(D)** The z-coordinate position of citrate's center of mass relative to the membrane center was calculated from the average position of POPC phosphorus atoms. **(A-D)** All distances are measured in Å as a function of simulation time.

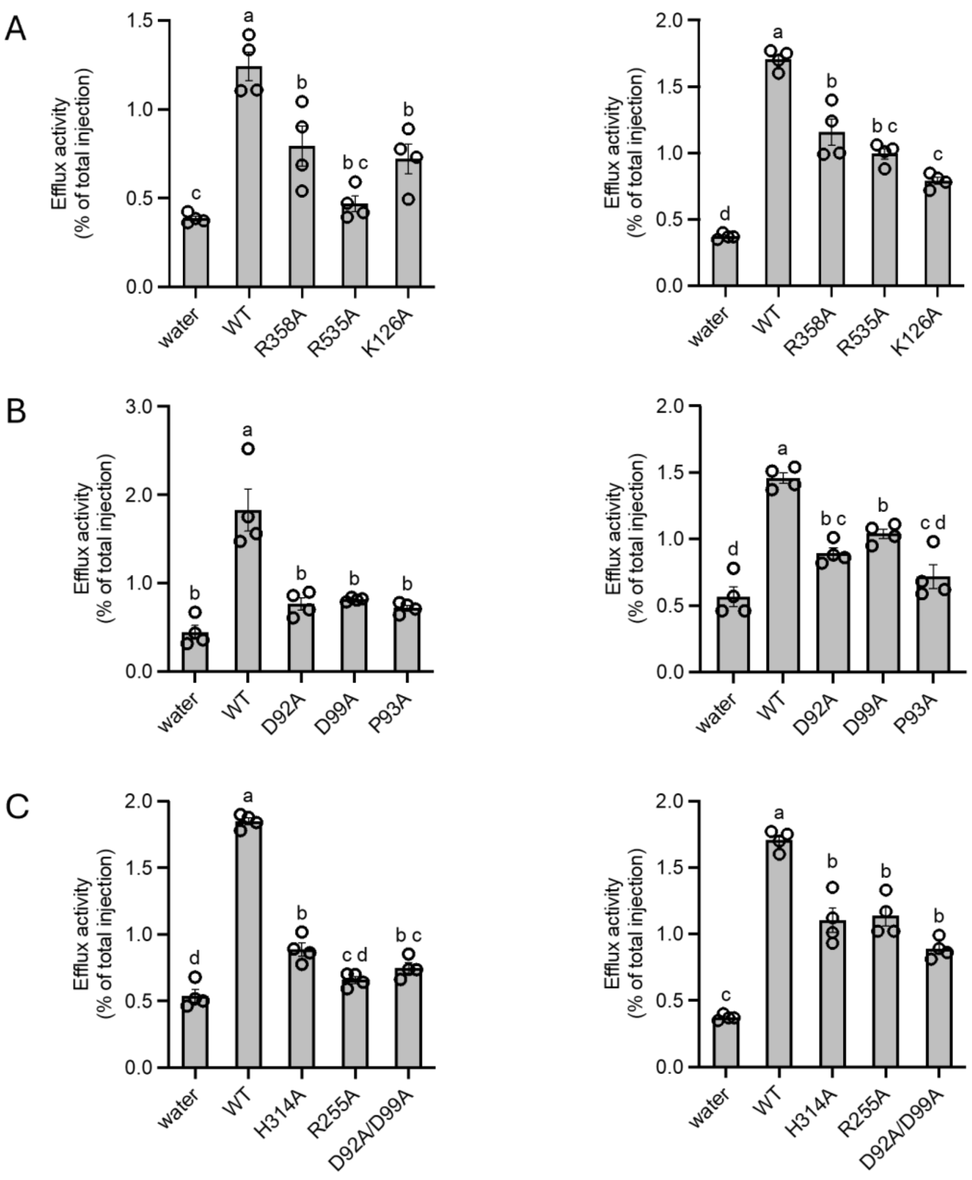

Fig. S19. Citrate efflux activity of HvACCT1 and its mutants expressed in *Xenopus oocytes*.

The impacts of mutations on **(A)** putative citrate binding sites, **(B)** potential protonation sites, and **(C)** the extensive hydrogen bond network in the N-lobe cavity, were examined. **(A-C)** The release of ^{14}C-labeled citrate was measured after 30 minutes. The experiments were conducted twice independently, which were shown in the right and left panels, respectively. Data are presented as means ± SEM (n=4, each replicate containing 4-6 oocytes). According to Tukey-Kramer's test, different letters indicate significant differences ($p < 0.05$).

Table S1. Crystallographic data collection and refinement statistics.

Data collection	HvAACT1$_{cryst}$	
Wavelength (Å)	1.0000000	
Resolution range (Å)	40 - 3.20 (3.39 - 3.20)*	
Space group	$P4_32_12$	
Unit cell	a=96.45 Å, b=96.45 Å, c=206.28 Å, α=β=γ=90°	
No. of unique reflections	16,789 (2,647)	
Completeness (%)	99.8 (99.4)	
R-factor (%)	7.7 (344.0)	
Multiplicity	7.7 (7.4)	
CC1/2	0.998 (0.543)	
Mean I/sima (I)	11.45 (0.47)	
**Refinement**	**Original Data**	**Data with truncation and anisotropic scaling**
Resolution (Å)	20 - 3.20 (3.30 - 3.20)	20 - 3.21 (3.41 - 3.21)†
No. of reflections	29,802 (5,023)	16,681 (211)
Completeness (%)	99.6 (98.2)	56.1 (4.2)
R-work (%)	28.93 (48.49)	26.00 (53.14)
R-free (%)	31.91 (46.62)	30.04 (61.67)
Willson B-factor (Å^2)	149.1	
No of non-H atoms	3,385	3,385
Macromolecules	3,385	3,385
Ligands and waters	0	0
Protein residues	455	455
Average B-factor (Å^2)	342.5	90.5
RMSDs Bond length (Å)	0.0041	0.0035
RMSDs Bond angles (°)	0.719	0.667
Ramachandran plot (%)		
Favored	99.78	99.56
Allowed	0.22	0.34
Disallowed	0.00	0.00
Clash score	9.5	10.5

*Values in parenthesis are those of the highest resolution shell.

†The resolution limits of the a*, b*, and c* axes were 4.3 Å, 4.3 Å, and 3.2 Å, respectively.

Table S2. Root mean square deviation (RMSD) between structurally reported MATE transporters and the structure of $HvAACT1_{cryst}$.

PDB	Protein name	RMSD (Å) of C_α		Notes on structure
		Outlier rejected*	All atoms	
7PHP	NorM-VC	1.901	3.723	Outward-facing
3VVO	PfMATE	2.203	4.328	Outward-facing (TM1 bent)
6Z70	Aq-126	2.931	3.196	Outward-facing
4LZ6	DinF-BH	2.717	3.703	Outward-facing
3VVN	PfMATE	2.999	4.304	Outward-facing (TM1straight)
5C6O	DinF-BH	3.845	3.845	Verapamil bound
6HFB	PfMATE	3.383	4.229	Outward-facing (TM1 straight)
7DQK	NtMATE2	3.833	4.348	Outward-facing
5Y50	AtDTX14	3.733	4.351	Outward-facing (TM7 bent)
5YCK	CasMATE	3.752	4.500	Outward-facing
4MLB	PfMATE	4.083	4.633	Outward-facing
6FHZ	PfMATE	6.543	10.855	Inward-facing

*Atoms with a large deviation (2-Å cutoff) from their aligned counterparts were considered outliers and rejected in default settings in PyMol.

Table S3. pK_a values of critical residues in HvAACT1

Residue	pK_a
D92	2.5
D99	5.2
K126	11.6
R255	>12
D289	7.3
D296	10.4
H314	3.1
R358	>12
D485	<0
R535	>12

Table S4. List of MATE proteins used in phylogenetic analysis

Group	Protein	UniProt /NCBI ID	Species	Substrates	PDB
**eMATE1**	AtDTX1	Q9SIA5	*Arabidopsis thaliana (At)*	Alkaloids, Cd^{+}, antibiotics, and other toxic compounds	
	AtDTX2	Q8GXM8	*At*	-	
	AtDTX3	Q9SIA4	*At*	-	
	AtDTX4	Q9SIA3	*At*	-	
	AtDTX5	Q9SIA1	*At*	-	
	AtDTX6	Q8RWF5	*At*	-	
	AtDTX7	Q1PFG9	*At*	-	
	AtDTX8	F4HQ05	*At*	-	
	AtDTX9	Q9C9M8	*At*	-	
	AtDTX10	Q8VYL8	*At*	-	
	AtDTX11	F4HZH9	*At*	-	
	AtDTX12	Q8L731	*At*	-	
	AtDTX13	Q94AL1	*At*	-	
	AtDTX14	Q9C994	*At*	-	5Y50
	AtDTX15	F4IHU9	*At*	-	
	AtDTX16	Q9FHB6	*At*	-	
	AtDTX17	Q9C9U1	*At*	-	
	AtDTX18	Q9LUH3	*At*	-	
	AtDTX19	Q9LUH2	*At*	-	
	NtJAT1	CAQ51477	*Nicotiana tabacum*	Nicotine	
	CasMATE	A0A2D0TCI5	*Camelina sativa*	-	5YCK
**eMATE2**	AtDTX20	F4HPH2	*At*	-	
	AtDTX21	Q8W488	*At*	-	
	AtDTX22	F4HPH1	*At*	-	
	AtDTX23	Q8RXK1	*At*	-	
	AtDTX24	F4J158	*At*	-	
	AtDTX25	Q8L616	*At*	-	
	AtDTX26	Q1PDX9	*At*	-	
	AtDTX27	Q9FKQ1	*At*	-	
	AtDTX28	Q9FNC1	*At*	-	
	AtDTX29	Q38956	*At*	-	
	AtDTX30	Q9LS19	*At*	-	
	AtDTX31	Q9LPV4	*At*	-	
	AtDTX32	F4I4Q3	*At*	-	
	AtDTX33	Q9SX83	*At*	-	
	AtDTX34	F4JH46	*At*	-	
	AtDTX35 /AtFFT	F4JTB3	*At*	-	
	AtDTX36	Q9SAB0	*At*	-	
	AtDTX37	O80695	*At*	-	
	AtDTX38	F4JKB9	*At*	-	
	AtDTX39	Q940N9	*At*	-	
	AtDTX40	Q9LVD9	*At*	-	
	AtDTX41 /AtTT12	Q9LYT3	*At*	Proanthocyanidins	
	OsPEZ1	XP_015632220	*Oryza sativa*	Phenolic compounds	
	OsPEZ2	BAG95442	*Oryza sativa*	Phenolic compounds	

	NtMATE1	BAF47751	*Nicotiana tabacum*	Nicotine	
	NtMATE2	BAF47752	*Nicotiana tabacum*	Nicotine	7DQK
	MdMATE1	ADO22709	*Malus domestica*	Proanthocyanidins	
	MdMATE2	ADO22711	*Malus domestica*	Proanthocyanidins	
	VvAM1	ACN91542	*Vitis vinifera*	Acylated anthocyanins	
	VvAM3	ACN88706	*Vitis vinifera*	Acylated anthocyanins	
	VvMATE1	XP_002282907	*Vitis vinifera*	Proanthocyanidins	
	VvMATE2	XP_002282932	*Vitis vinifera*	Proanthocyanidins	
	MtMATE1	ACX37118	*Medicago truncatula*	Proanthocyanidins	
	MtMATE2	HM856605	*Medicago truncatula*	Proanthocyanidins	
**eMATE3 /CATE-MATE**	AtDTX42 /AtMATE	Q9SYD6	*At*	Citrate	
	AtDTX43 /AtFRD3	Q9SFB0	*At*	Citrate	
	AtDTX44	Q84K71	*At*	-	
	AtDTX45	Q9SVE7	*At*	-	
	AtDTX46	Q8W4G3	*At*	-	
	AtDTX47 /AtEDS1	Q945F0	*At*	Salicylic acid	
	BoMATE	AGU16976	*Brassica oleracea*	Citrate	
	EcMATE	BAM68467	*Eucalyptus camaldulensis*	Citrate	
	EcMATE4	BAM68468	*Eucalyptus camaldulensis*	Citrate	
	GmFRD3a	ACE89001	*Glycine max*	Citrate	
	HvAACT1	BAF75822	*Hordeum vulgare*	Citrate	
	OsFRDL1	BAG95121	*Oryza sativa*	Citrate	
	OsFRDL2	BAG95152	*Oryza sativa*	Citrate	
	OsFRDL4	BAL41687	*Oryza sativa*	Citrate	
	MtMATE66	AMP17769	*Medicago truncatula*	Citrate	
	MtMATE69	AMP17768	*Medicago truncatula*	Citrate	
	TaMATE1b	AFZ61900	*Triticum aestivum*	Citrate	
	SbMATE	ABS89149	*Sorghum bicolor*	Citrate	
	VuMATE1	AIS76465	*Vigna umbellata*	Citrate	
	ZmMATE1	ACM47309	*Zea mays*	Citrate	
	AbMATE	MDJ0498295	*Acidimicrobiia bacterium*	-	
	BbMATE	RDV38435	*Bradymonadaceae bacterium*	-	
	GbMATE	MDH3296898	*Gemmatimonadota bacterium*	-	
	LsMATE	WP_127780844	*Lujinxingia sediminis*	-	
	AvMATE	B9JR04	*Agrobacterium vitis*	-	
**eMATE4**	AtDTX48	Q9SLV0	*At*	-	
	AtDTX49	O82752	*At*	-	
	AtDTX50	Q9FJ87	*At*	Abscisic acid	
	AtDTX51	Q9SZE2	*At*	-	
	AtDTX52	Q4PSF4	*At*	-	
	AtDTX53	Q9ZVH5	*At*	-	

	AtDTX54	Q9LE20	*At*	-	
	AtDTX55	Q9FH21	*At*	-	
	AtDTX56	O49660	*At*	-	
**NorM subfamily**	NorM-NG	4HUN	*Neisseria gonorrhoeae*	Cs^+, Ethidium, Rhodamine-6G	4HUL, 4HUM, 4HUN, 5C6P
	NorM-VC	3MKT	*Vibrio cholerae*	Drugs and toxic compounds	7PHP, 3MKT
	NorM-VP	BAA31456	*Vibrio parahaemolyticus*	Drugs and toxic compounds	
	MdtK	Q7WTR3	*Erwinia amylovora*	Drugs and toxic compounds	
**DinF subfamily**	DinF-BH	Q9KAX3	*Bacillus halodurans*	Ethidium, Rhodamine-6G	4LZ6
	PfMATE	Q8U2X0	*Pyrococcus furiosus*	Ethidium, Rhodamine-6G	3VVO, 3VVN, 3VVR, 4MLB, 6HFB, 6FHZ
	VcmN	C3LWQ2	*Vibrio cholerae*	Drugs and toxic compounds	6IDP, 6IDR
**aMATE1**	Aq_128	O66528	*Aquifex aeolicus*	Drugs and toxic compounds	6Z70

Dataset S1 (separate file). Source data for functional assays.
